# Scalable decision-making for games of imperfect information

**DOI:** 10.1038/s41586-026-11036-y

**Published:** 2026-09-30

**Authors:** Samuel Sokota, Eugene Vinitsky, Hengyuan Hu, Zhiyuan Fan, J. Zico Kolter, Gabriele Farina

**Affiliations:** 1 Machine Learning Department, Carnegie Mellon University, Pittsburgh, PA USA; 2 Department of Civil, Urban, and Environmental Engineering, New York University, Brooklyn, NY USA; 3 Department of Computer Science, Stanford University, Stanford, CA USA; 4 Department of Electrical Engineering and Computer Science, Massachusetts Institute of Technology, Cambridge, MA USA

**Keywords:** Computer science, Computational science

## Abstract

Real-world decision-making generally involves hidden information, that is, information that is unknown to one agent but possessed by another. Unfortunately, the presence of large amounts of hidden information renders established reinforcement learning and search approaches ineffective. Even with multimillion-dollar industrial research efforts^1^, top-human-level play at Stratego—a board wargame with hidden information on a massive scale—has remained beyond the reach of artificial intelligence (AI). Here we introduce Ataraxos, an AI for Stratego based on general techniques that we developed for both self-play reinforcement learning and test-time search under hidden information. Ataraxos defeated the most decorated human Stratego player of all time by a large margin—achieving, to our knowledge, the first superhuman result in the game’s history—while consuming orders of magnitude less compute and data than previous efforts. Using the same techniques, we built a superhuman AI for Barrage Stratego and state-of-the-art AIs for Hanabi and *dou dizhu*, all with low cost and high sample efficiency. The success of this approach across adversarial, cooperative and team games establishes a design pattern for reinforcement learning and search that is effective under large amounts of hidden information, a longstanding desideratum of the field of strategic decision-making.

## Abstract (web summary)

Ataraxos, an AI for the board wargame Stratego, establishes a design pattern for reinforcement learning and search that is effective under large amounts of hidden information, a longstanding desideratum of the field of strategic decision-making.

## Main

Imperfect information^2,3^ is a term of art describing interactions in which some agents may possess information that others do not. It is a characterizing feature of real-world settings, including financial markets, military conflict and negotiations.

**Fig. 1:**
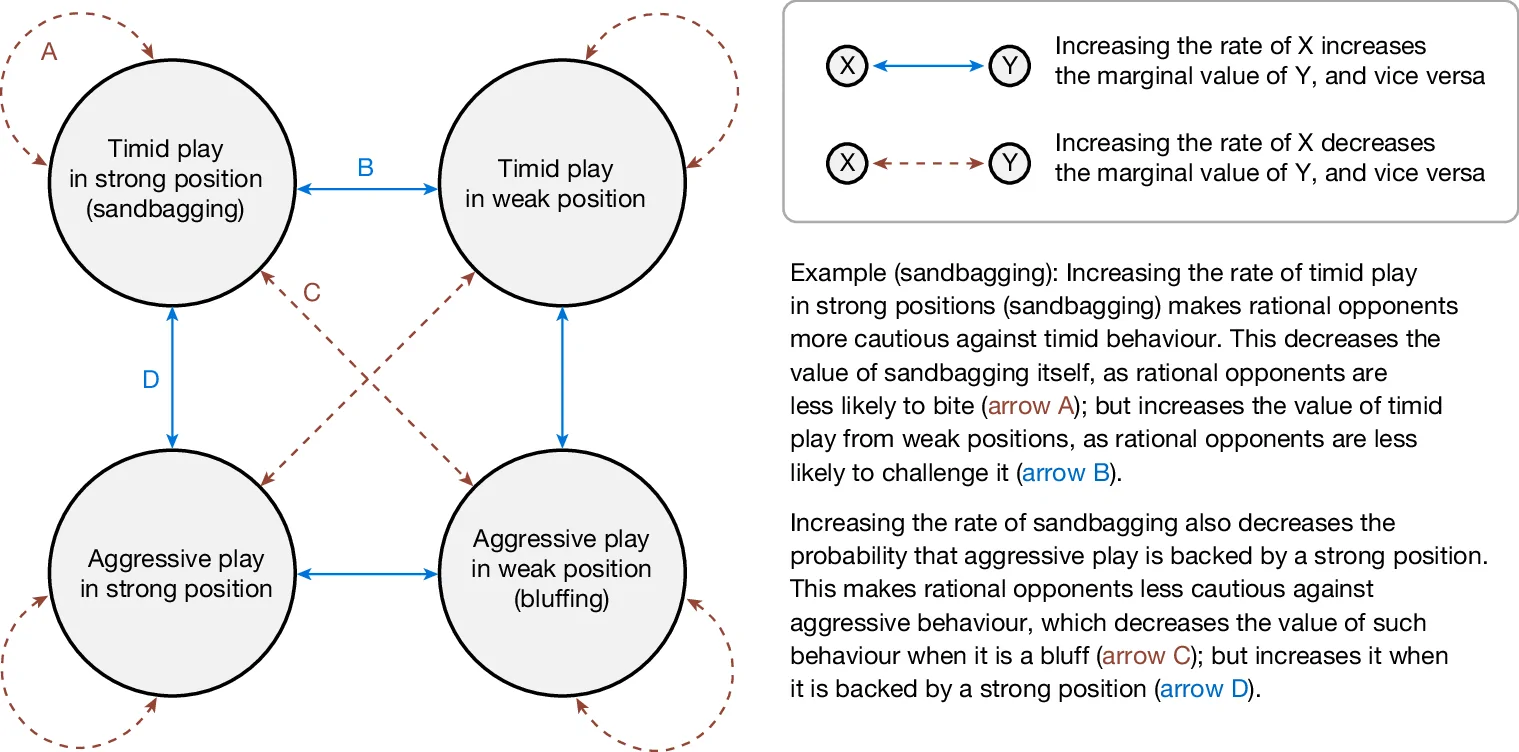
Graphical representation of differential relationships between decision frequencies and values among contemporaneous counterfactuals under imperfect information. The nodes represent decisions. The colour and line style of an edge represent whether changing the frequency of a decision on one end of the edge increases (blue and solid) or decreases (brown and dashed) the marginal value of the decision on the other end of the edge. The interconnectedness of frequencies and values across decisions makes it difficult to intuit the frequencies with which decisions should be made.

Whereas in perfect-information settings, such as chess and Go, the conceptual ‘right thing to do’ is simple (select a move that maximizes the value of the resulting position), in those of imperfect information, it is subtle. The value of a decision depends not only on the policies agents employ thereafter but also on those they used—and counterfactually would have used—before and at the time of the decision (as these all affect the posterior distribution over hidden information). Even reasoning about the dependencies among contemporaneous counterfactuals in isolation is complex (Fig. 1).

These dependencies have made developing artificial intelligence (AI) for imperfect-information settings challenging. The most successful approaches use sophisticated problem transformations based on public information^4–11^. But the cost of these transformations scales with the amount of hidden information, making them applicable only when this amount is small, as in Texas hold’em (in which there are 1,326 possible hands). Owing to this fundamental limitation, and the absence of an alternative foundation, strategic decision-making in settings with large amounts of hidden information has remained an open problem.

**Fig. 2:**
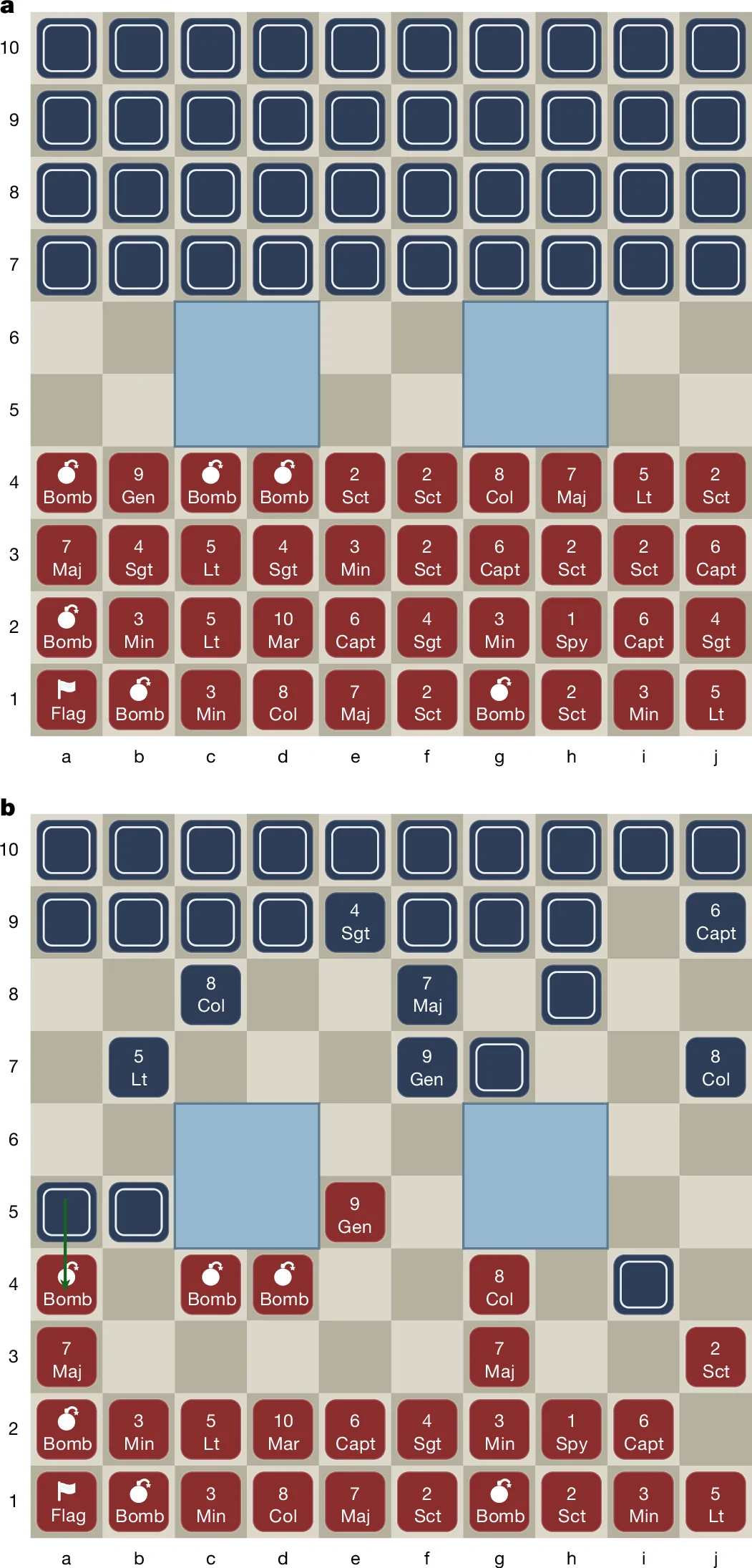
An illustration of the game of Stratego. **a**, The starting position of a game from the perspective of the red player. Each player arranges their pieces in secret, so the types of the blue pieces are concealed from the red player (and vice versa). From this starting position, the players alternate moves, each of which is defined by the displacement of a single piece, as in chess. **b**, A move in the game for the blue player. On this move, because a blue piece moves onto a square occupied by a red piece, there is a battle. During a battle, the types of both pieces are revealed and at least one of the two is captured (determined based on their types and attack–defence roles). If a Flag (see piece on a1) is captured in battle, the capturing player wins the game.

The unresolvedness of this problem is epitomized by the state of AI for Stratego—a board wargame resembling military chess (Fig. 2) prized as a challenge problem for the scale of its hidden information (there are over 10^33^ possible piece configurations). Stratego has been the subject of industrial research efforts spanning multiple years, involving dozens of researchers, and expending computation costing millions of dollars^1^. Yet, human players have remained superior, potentially making it the only classical game in which such well-resourced efforts have failed to produce superhuman performance.

Here we introduce Ataraxos, an AI for Stratego. Ataraxos was built using general techniques that we developed for self-play reinforcement learning and test-time search under massive amounts of hidden information. In a 20-game series, Ataraxos defeated Pim Niemeijer—the most decorated Stratego player of all time—by a margin of victory without precedent at the highest level of play: 15 wins, 1 loss and 4 draws. Ataraxos achieved this result while costing only a few thousand dollars to train.

To demonstrate generality, we applied these techniques to build AIs for Barrage Stratego, a Stratego variant; Hanabi, the premier benchmark for cooperative imperfect-information games; and *dou dizhu*, one of the most popular card games in China. Our AIs defeated three multi-time world champions in Barrage Stratego (to our knowledge, the first superhuman result for the game), achieved a new state of the art for Hanabi, and beat the state-of-the-art bots for *dou dizhu*.

The success of these techniques across adversarial, cooperative and team games shows that reinforcement learning and search are no longer precluded from high performance by the presence of large amounts of hidden information. This marks the fulfilment of a longstanding aspiration of the field of strategic decision-making.

## Overview of Ataraxos

Ataraxos is based on *tabula rasa* self-play reinforcement learning and test-time search—a design pattern that is general across imperfect-information games. The pattern comprises a policy–value network, trained through self-play to propose decisions and predict game outcomes; a belief network, trained on self-play data to model hidden information; and a search procedure that refines the policy at test time.

In self-play reinforcement learning, the policy–value network improves by playing games against itself and shifting probability towards decisions that lead to wins. The core innovation that enables effective reinforcement learning is a coordination between regularization strength and policy update size^12^. Ataraxos uses stronger regularization and more aggressive updates early in self-play, and weaker regularization and smaller policy updates late in self-play. Two complementary considerations motivate this interplay. First, keeping update sizes commensurate with regularization strength damps the otherwise cyclical, divergent or chaotic learning dynamics engendered by imperfect information, leading to stable progress. Second, adapting both quantities over the course of training facilitates rapid improvements early on and continued improvement later—avoiding both update sizes so small as to make improvement impractical and regularization so large as to compromise the policy.

Ataraxos trains a belief network on the self-play games of its final policy–value network. Given the information available to the player at a position, this network is trained to model the hidden information—whose ground truth is available in self-play. This trained belief network serves as a generative model, allowing Ataraxos to sample realizations of hidden information based on their likelihoods.

At test time, Ataraxos selects its actions by search. To start, it generates realizations of hidden information with its belief network. Then, with its policy–value network, it plays out candidate actions under each realization and estimates the values of these actions from the positions reached. Finally, treating these estimates as if they had come from self-play, Ataraxos performs one additional update step—applied only to the current decision—and selects its action from the updated policy. Because this step mimics those of self-play reinforcement learning, the search inherits improvement properties thereof^13^.

For Stratego, Ataraxos instantiates two interdependent self-play processes, corresponding to the two phases of the game: in one, a set-up network learns to arrange the 40 pieces; in the other, a move network learns to move the pieces. The two processes are coupled through the games themselves—the set-up process supplies the initial boards on which the move process plays, and the outcomes of the resulting games drive the updates of both. The belief network learns to predict the types of the opponent’s hidden pieces. At test time, set-ups are sampled directly from the set-up network, while moves are selected using search.

We trained the resulting Stratego system at a total cost of a few thousand dollars. This economy reflects both a fast implementation—a custom simulator that uses NVIDIA’s Compute Unified Device Architecture (CUDA)^14^ to execute millions of state updates per second—and sample-efficient learning.

## Evaluation

We evaluated Ataraxos against Pim Niemeijer^15,16^ (hereafter Pim), a player whose accolades include:

- 4 world championships (the most of any active player and tied for the most of all time)
- 15 Dutch national championships (the most of all time)
- 2 online world championships (the most of all time)
- Over 600 weeks as the number-1-ranked player (the most of all time).

According to George Franka^17^, the only player to compete in every world championship since 1997, Pim is “the best Stratego player ever”.

The evaluation consisted of a 20-game series, with performance measured by effective win rate (that is, by counting draws as half wins). The large number of games was chosen both to reduce variance (because strong play in Stratego requires substantial randomization, competent players win games against top players more often than they would in games such as chess) and to give Pim an opportunity to find and exploit weaknesses in the strategy of Ataraxos—which, he was informed, would not adapt to his play. To prevent fatigue and allow time for tactical preparation between games, the evaluation was spread over the course of 3 weeks. Pim was paid US$1,000 for participating in the evaluation, with an additional US$100 for each win and US$50 for each draw to align his monetary incentives with his performance.

**Fig. 3:**
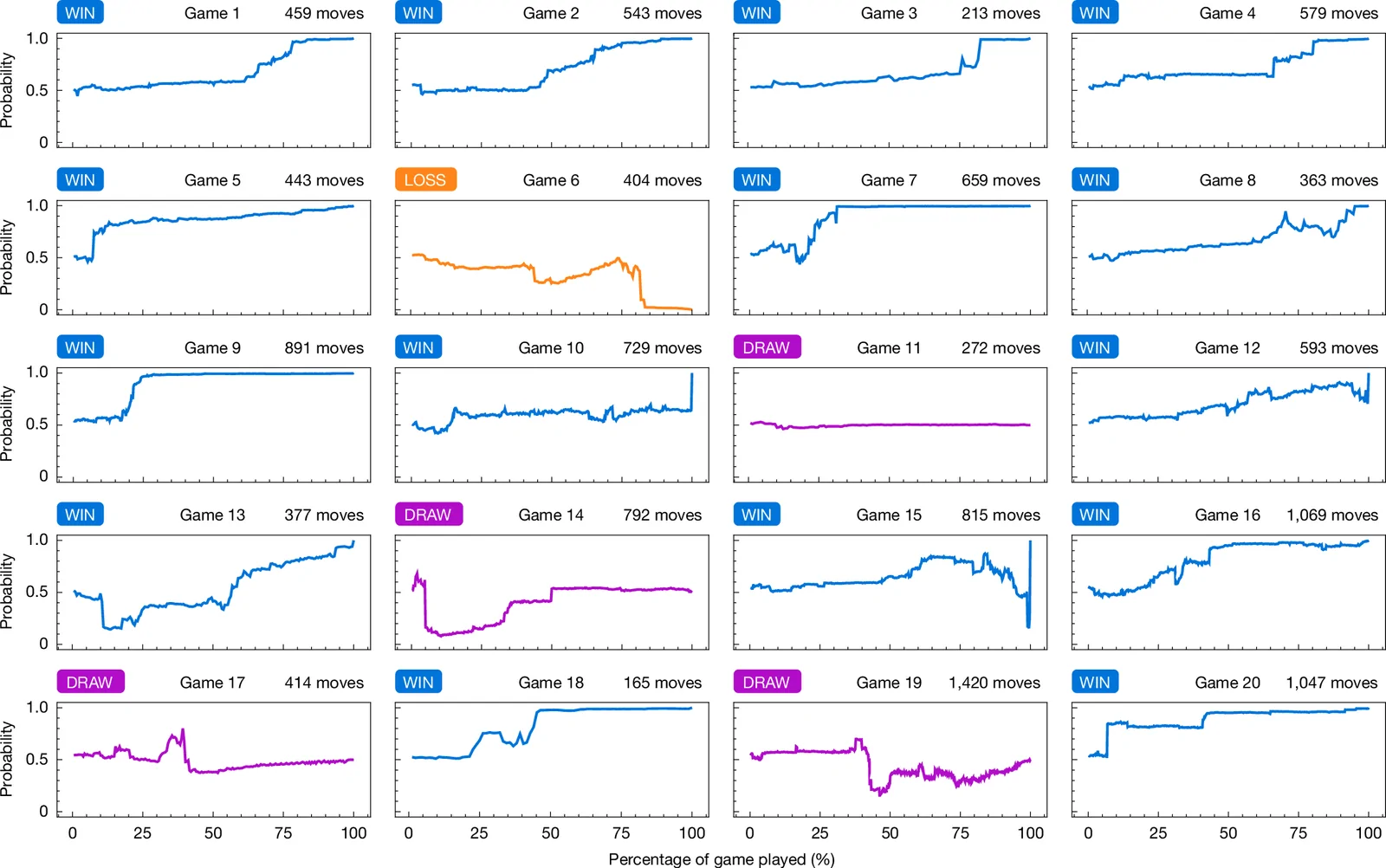
Effective win probability implied by the network value predictions over the games played against Pim Niemeijer (*n *= 20). Effective win probability is the expected value of the game with winning assigned value 1, drawing assigned value 1/2 and losing assigned value 0.

Ataraxos won the series with 15 wins, 1 loss and 4 draws (Fig. 3). This margin (an 85% effective win rate) is without precedent at the highest level of human play, where, according to three-time runner-up world champion Max Roelofs^18^, margins are razor thin owing to the risk that must inevitably be assumed during play. Ataraxos achieved such a margin despite the asymmetric structure of the evaluation—wherein Pim could adapt to Ataraxos over a large number of games, but Ataraxos could not adapt to Pim—which, according to three-time world champion Vincent de Boer^19^, constituted a large handicap.

Because of this adversarial adaptation, and more broadly because human strategies are not static from game to game, the outcomes of the games were far from independently and identically distributed. But under the assumption that they had been, an exact one-sided binomial test of whether Ataraxos was more likely to win than lose would have yielded a *P* value less than 2.6 × 10^−4^.

Following the evaluation against Pim, we demoed Ataraxos at the 2025 Stratego World Championship from 1 to 3 August. During the demo, world championship attendees were given the opportunity to play against Ataraxos. Across 40 such games, Ataraxos recorded a 95% effective win rate (38 wins, 2 losses, 0 draws), providing additional evidence of strength against a range of players and playing styles.

## Application to other games

We applied the techniques underlying Ataraxos to build AIs for Barrage Stratego, Hanabi and *dou dizhu* (each of which we also refer to as Ataraxos, after their shared design), thus spanning two-player zero-sum, fully cooperative and two-team zero-sum games. In all cases, we achieved state-of-the-art results, illustrating the effectiveness of these techniques across a diverse collection of imperfect-information settings.

### Barrage Stratego

Barrage Stratego is a competitively played variant of Stratego involving 8 pieces per player (as opposed to the classic variant, which involves 40). Games of Barrage are more precarious than those of the classic variant, and good strategy requires a higher degree of risk taking, bluffing and sandbagging.

We evaluated our AI for Barrage Stratego against three of the four top-ranked human players, each of whom is a two-time Barrage World Champion. The evaluations consisted of four 50-game series (one each against the number-3- and number-4-ranked players and two against the number-1-ranked player). We used a large number of games both because games of Barrage are typically shorter than those of classic Stratego and because individual game outcomes tend to be more stochastic than in the classic variant.

Ataraxos won each of the four series. With the same caveats and under the same assumptions as in the ‘Evaluation’ section, an exact one-sided binomial test of its aggregate record would have yielded a *P* value less than 1.3 × 10^−5^. To our knowledge, these results are the first in which AIs have outperformed top humans at Barrage Stratego.

### Hanabi

Hanabi is the most popular benchmark among fully cooperative imperfect-information games^20^ and the winner of the 2013 Spiel des Jahres award for best board game of the year. In the game, players jointly build five colour-coded fireworks stacks in ascending order, each player holding their cards facing outwards so that only teammates can see them. On each turn, a player either reveals limited information about the cards of a teammate, discards a card or attempts to extend one of the stacks, with the final score determined by the number of cards successfully played. The number of deals exceeds 5 × 10^27^ for the five-player variant.

We implemented Ataraxos for each of the two- to five-player variants of the game. Ataraxos set a new state of the art with statistical significance for all variants. For the two-player variant, our result required two orders of magnitude less compute than the previous state of the art.

### *Dou Dizhu*

*Dou dizhu* is one of the most popular card games in China, with millions of active players and mobile apps amassing billions of downloads^21,22^. One player, the landlord, competes against a team of two peasants in a three-player, asymmetric partnership game. The landlord begins with a larger hand and plays alone, while the two peasants cooperate implicitly through their actions. The objective is to be the first to empty one’s hand by playing legal card combinations of progressively higher rank, with players alternating turns and either beating the current combination or passing. The number of possible deals in the game exceeds 3 × 10^24^.

We measured the performance of our approach against the previous state of the art, PerfectDou^23^, as well as its predecessor DouZero^24^. Following PerfectDou, the evaluation treated the game as a player-versus-team setting, where one agent controls the landlord and the other controls both peasants in a decentralized fashion. Ataraxos defeated PerfectDou and DouZero with statistical significance, establishing a new state of the art.

## Conclusion

The combination of reinforcement learning and search has proven to be a powerful recipe for strategic decision-making^25–31^. But in the past, this recipe has had limited applicability to imperfect-information settings. The success of the AIs presented herein shows that the presence of large amounts of hidden information is no longer prohibitive. This puts practical AI within reach for many strategic decision-making problems for which fast, accurate simulators can be constructed.

## Methods

### Design of Ataraxos

Ataraxos consists of two interdependent self-play reinforcement learning processes, realized by transformer networks for set-up selection and move selection; a belief network trained on self-play games of the final selection networks; and a test-time search procedure that composes the move and belief networks.

#### Interdependent self-play processes

The foundation of Ataraxos is its self-play reinforcement learning module. This module comprises two separate but interdependent self-play processes associated with the two phases of Stratego. The first phase, in which players privately determine starting positions for their pieces—called set-ups—is handled by one self-play process. The second phase, in which players alternate moving their pieces, is handled by another. These processes learn separately but in tandem: the set-up selection process determines the initial boards for the move selection process; and the move selection process determines game outcomes that both processes use for policy updates. We chose this decomposition rather than an end-to-end model because, although an end-to-end approach would avoid making the phase decomposition explicit, it would require a single model and training pipeline to accommodate two unrelated tasks. As discussed in the sections ‘Reinforcement learning with transformers’, ‘Self-play training data generation’ and ‘Dynamically damped self-play’, with full Stratego training specifications provided in Supplementary Information, set-up learning favoured a decoder-only architecture, Monte Carlo estimation of expected returns and advantages, and higher learning rates and regularization temperatures. Move learning, by contrast, favoured an encoder-only architecture, *λ*-based expected return and advantage estimators (in which *λ* controls the weighting of multi-step estimates), an annealed learning-rate schedule, and lower regularization temperatures.

#### Reinforcement learning with transformers

**Extended Data Fig. 1:**
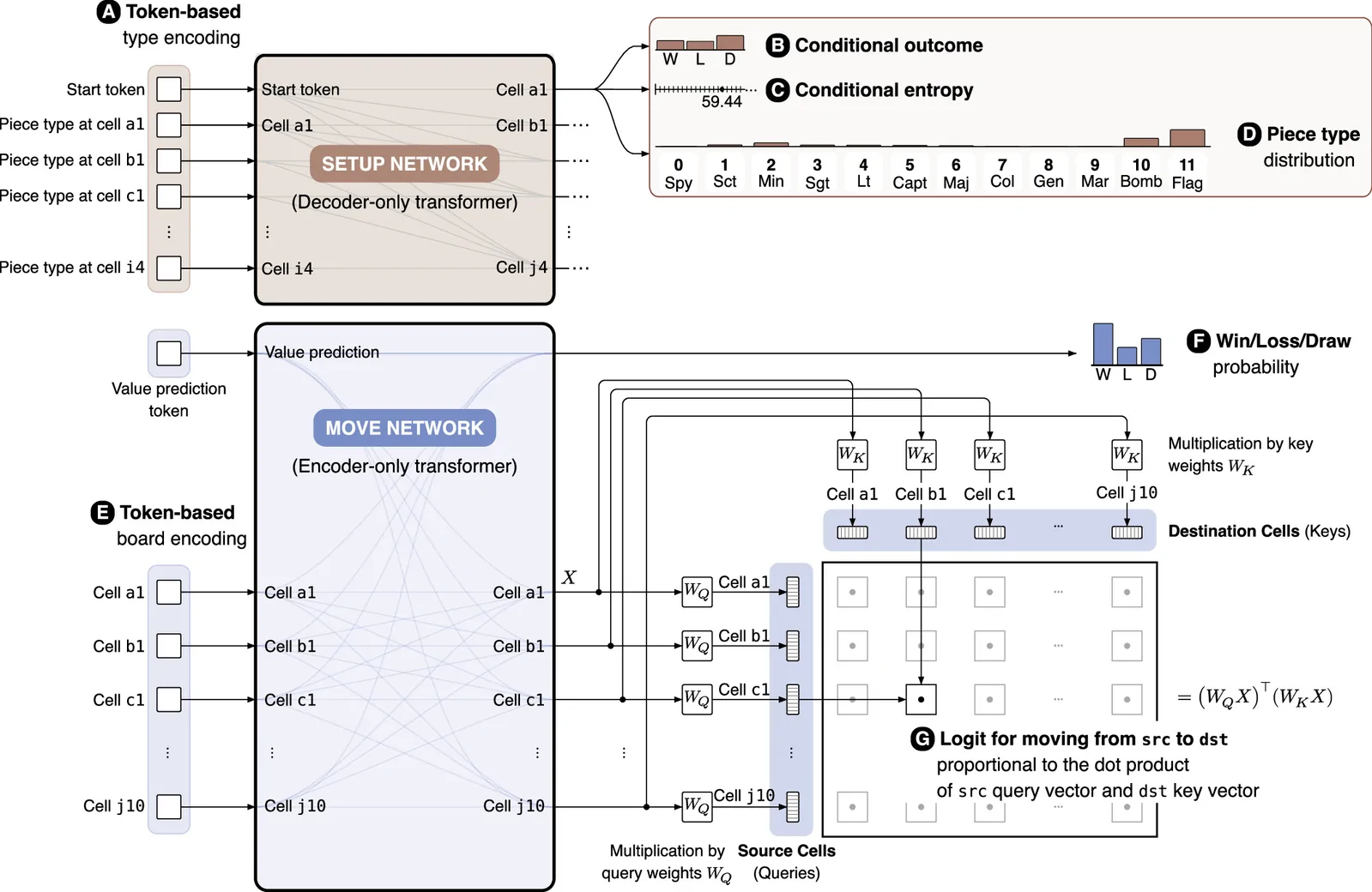
Set-up and move network diagrams. **Top**, the set-up network is a decoder-only transformer that generates set-ups by autoregressively placing the 40 pieces onto squares of the board in row-major order (i.e., first row, first column; first row, second column; and so forth). (**A**) As input, the set-up network takes tokenized piece types with learned absolute positional embeddings^34^. As output, for each set-up prefix, the set-up network provides: (**B**) an estimate of the conditional probability of winning, losing, or drawing a self-play game; (**C**) an estimate of the conditional entropy of the set-up; and (**D**) probabilities for placing pieces of each type on that square. **Bottom**, the move network is an encoder-only transformer that selects the piece to move and the square to which to move it. (**E**) As input, the move network takes tokenized representations of the occupiable squares with learned absolute positional embeddings, and an additional token for value prediction. The move network outputs: (**F**) a categorical prediction of the game outcome; and (**G**) probabilities for the moves computed using a key–query matrix product^33^.

To represent the policies and value functions for the two self-play processes, Ataraxos uses two transformers^32^, which we call the set-up network and the move network. These networks are diagrammed in Extended Data Fig. 1. Important choices included: parameterizing the set-up network as a decoder-only transformer, which allowed training on entire set-ups with single forward–backward passes; parameterizing the policy over moves using a key–query matrix product^33^, which learned faster than less sophisticated parameterizations; using learned absolute positional embeddings^34^ in both networks; and sizing the move network to balance sample efficiency against iteration speed, between which we observed substantial trade-offs.

#### Self-play training data generation

To generate self-play games, Ataraxos selects its set-ups and moves by directly sampling from the associated networks.

For the moves played during these games, Ataraxos computes the estimates of expected cumulants^35^ and advantages needed for training updates using *λ* returns^36,37^ (with distinct *λ* values), and trains only on the moves with large estimated advantage magnitudes^38^. Filtering in this way reduced the overall wall-clock time per reinforcement learning iteration by a factor of about 2.5, while simultaneously—in a phenomenon meriting further investigation—actually increasing both sample efficiency (with respect to the number of environment queries) and asymptotic performance.

For the set-ups, Ataraxos uses Monte Carlo returns (that is, final outcomes of games played by the current policy) to estimate these quantities, and does not use filtering. The superiority of Monte Carlo returns for advantage estimation is unusual in reinforcement learning, although similar behaviour has also been observed in language model reasoning^39^.

#### Dynamically damped self-play

Ataraxos trains on on-policy or close to on-policy self-play data by dynamically damping its learning dynamics. This allows it to leverage standard policy optimization tools to regularize and control the size of its policy updates, obviating the need for more onerous techniques, such as trajectory importance reweighting and policy averaging, for handling imperfect information.

For regularization, Ataraxos incorporates additional terms into the losses of its networks, as detailed in ‘Stratego implementation details’ in Supplementary Information. For the set-up network, it uses a maximum entropy term^40^; for the move network, it uses a myopic reverse Kullback–Leibler penalty towards the policy that selects a movable piece uniformly at random and then selects a legal move for this piece uniformly at random. Ataraxos anneals the coefficients of these regularization terms according to distinct power laws over training^12^. We found that this regularization functioned somewhat analogously to an energy reserve—annealing too cautiously left the playing ability of the model underdeveloped, while annealing too aggressively produced rapid initial gains but also collapsed the entropy of the model, depleting its capacity to learn thereafter and often making it easy to exploit.

For update size control, Ataraxos uses four mechanisms, which we found offered complementary benefits: a reverse Kullback–Leibler penalty to the data collection policy, importance ratio clipping^41^, gradient norm clipping^42^ and the learning rate of Adam^43^. Ataraxos anneals the learning rate for its move network according to a power law over the course of training. We found that scheduling this learning rate was crucial both for rapid learning early in training and for preventing plateauing later in training.

#### Belief modelling

To facilitate search, Ataraxos trains a belief network on every position of trajectories sampled from its final self-play policy. Given the information available to the player at a position, this network is trained with teacher forcing to maximize the log-likelihood of the true types of the opponent’s hidden pieces. The belief network processes the known information using a transformer encoder (similar to the move network) and predicts the types of unknown pieces autoregressively in row-major order using a transformer decoder. The resulting architecture is diagrammed in Extended Data Fig. 4. Ataraxos applies dropout^44^ to the belief network during training to aid generalization to out-of-distribution positions reachable by opponents that play very differently from its set-up and move networks, such as humans.

#### Test-time search via update equivalence

Given move and belief networks, the search Ataraxos uses is straightforward—it simply performs an additional damped self-play reinforcement learning update^13^. That such search is feasible at all, and moreover simple, is noteworthy, as test-time search in settings with as much hidden information as Stratego has been viewed as such a major technical challenge that previous work effectively forwent it^1^.

Ataraxos uses the belief network before each move to sample a collection of possible game states given its current position. For each candidate move, it runs depth-limited rollouts from these game states—starting with that candidate, then using its move network to simulate both players. Ataraxos estimates the value of the candidate move by averaging its network value predictions across the positions reached by rollouts starting from that move. These averaged values approximate self-play action values regardless of the policy of the opponent Ataraxos is facing, as the belief network approximates the posterior distribution of the self-play policy, the rollouts are executed by the self-play policy and the network predicts self-play position values.

Ataraxos uses these values to update its policy with a tabular step of magnetic mirror descent^12^, which regularizes and controls the size of the update with the same two reverse Kullback–Leibler divergences used during training. Importantly, this update can safely be more aggressive than those during training, both because the test-time update is tabular (and thus does not interfere with the policy at other positions), and because it is based on the more accurate advantage estimates enabled by the larger amount of computation per position at test time. The move Ataraxos plays is sampled from the updated policy. Set-ups, by contrast, are generated by sampling directly from the set-up network, with neither search nor any other intervention applied.

#### Training information

**Extended Data Fig. 2:**
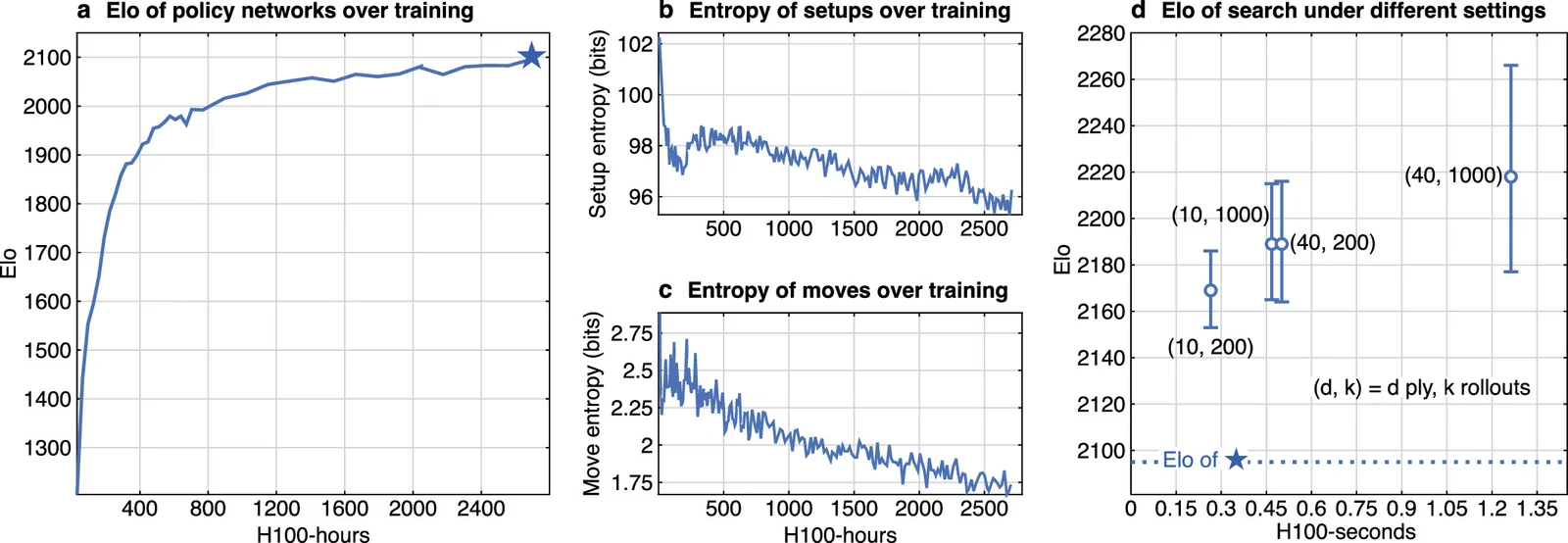
Performance and entropy of Ataraxos as a function of compute. **a** Elo of the policy networks over the course of training, as measured by performance against a fixed reference opponent. Quantifying skill level in Stratego by Elo is useful but flawed, as randomization can compress margins (i.e., make margins between two players smaller than would be predicted by their respective margins against a common third player whose skill level lies between them), among other reasons. Each Elo estimate was computed from *n* = 16,000 games against the fixed reference opponent. **b** The entropy of the set-ups and **c** the entropy of the moves (in expectation over the self-play distribution of positions). Dynamic damping shepherds these entropies smoothly downward over training, preventing them from collapsing. **d** Elo of search on a single NVIDIA H100 graphics processing unit under varying rollout counts and search depths with 95% confidence intervals. Deeper searches with more rollouts produce stronger performance but require more time per move. During the evaluation, Ataraxos used a 40-ply 1,000-rollout search that averaged about 1.26 seconds per move—a pace of play faster than that of human players.

The reinforcement learning training run utilized 16 NVIDIA H100 graphics processing units (GPUs) for 1 week; the training run for the belief network utilized 4 such units for 4 days. Extended Data Fig. 2 shows metrics for this training run and searches performed on top of its final networks.

The reinforcement learning training run comprised 163 million finished games, 208 billion environment steps, 8.56 million gradient steps for the move network and 99.5 thousand gradient steps for the set-up network.

Compared with previous work^1^—which did not reach the level of top humans—our reinforcement learning training run consumed roughly 1/500th of the compute cost, 1/30th of the self-play games and 1/100th of the training examples. Taken together with the step change in playing strength reported in the main text, the reductions in self-play games and training examples indicate that the lower training cost reflects not only faster implementation but also markedly greater sample efficiency.

### Rules of Stratego

#### Game summary

**Extended Data Fig. 3:**
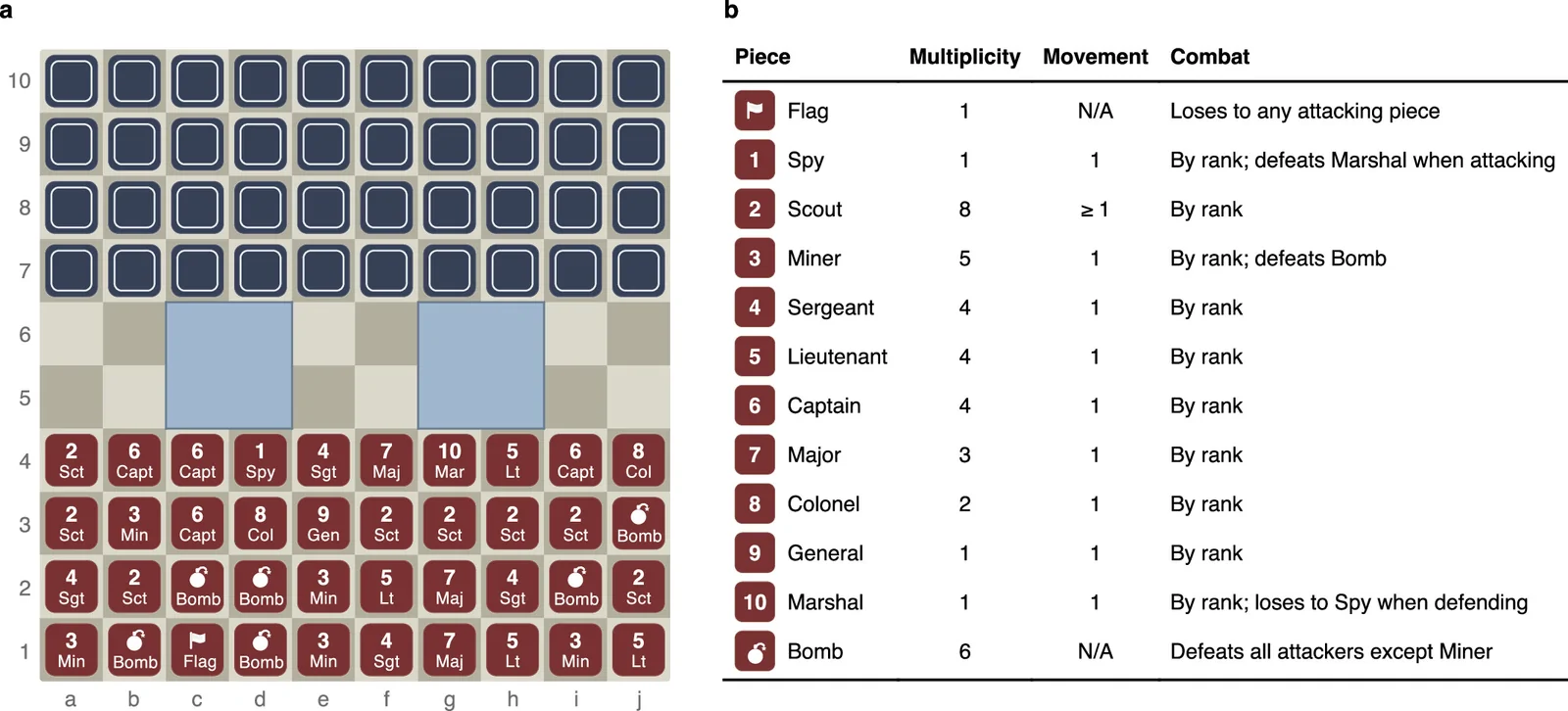
Stratego board layout and piece mechanics. **a** An example starting position. The light blue blocks in the middle of the board are called lakes and cannot be occupied by the pieces of either player. **b** Multiplicities, movement abilities, and combat rules for Stratego pieces. Scouts can move an arbitrary number of squares in cardinal directions; other movable pieces can move a single square in cardinal directions. *By rank* means that the higher numbered piece defeats the lower numbered piece.

**Extended Data Fig. 4:**
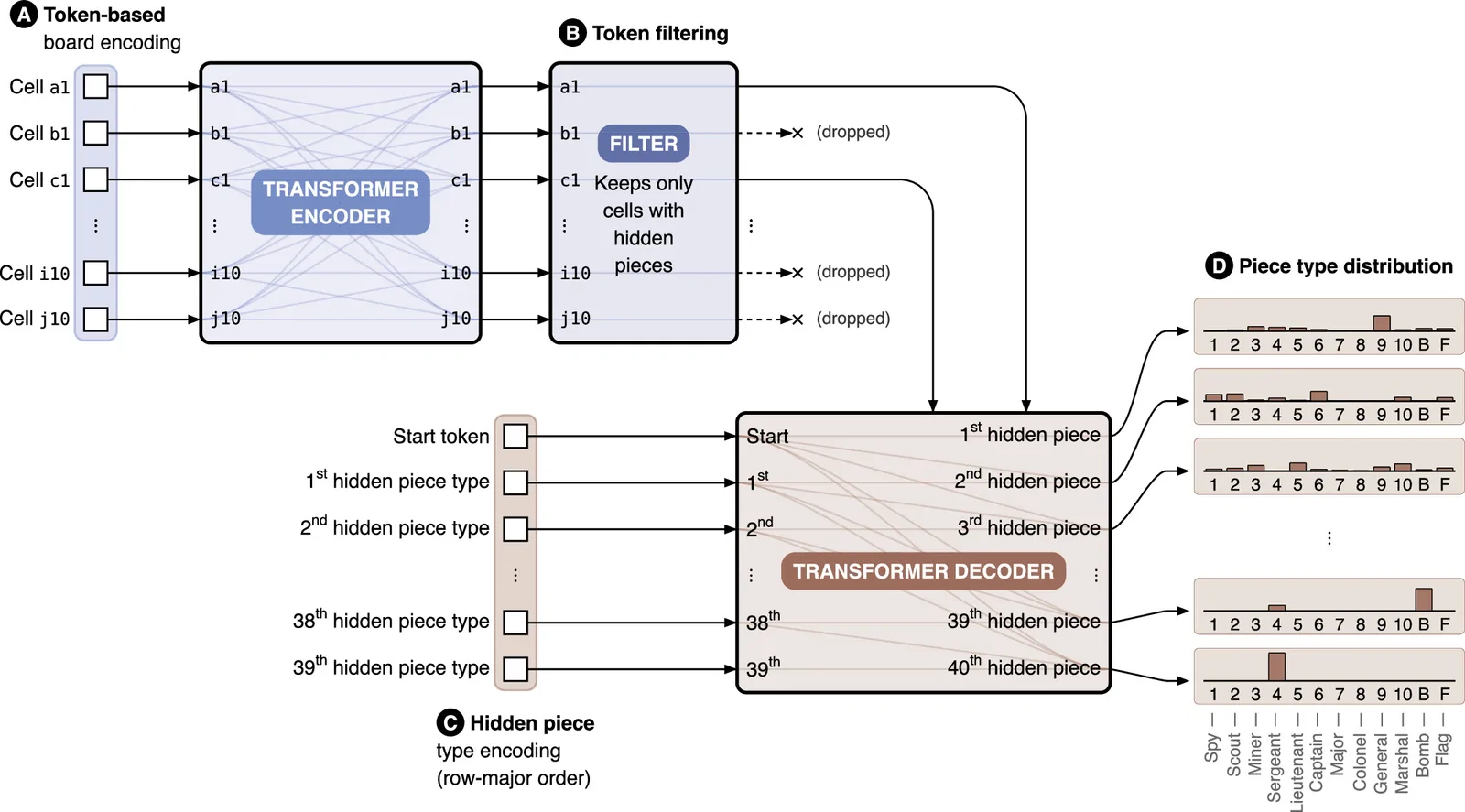
Diagram of belief architecture. (**A**) The input to the encoder is a tokenized representation of the occupiable squares with learned absolute positional embeddings, similar to the move network. (**B**) The output of the encoder is filtered to keep only tokens corresponding to squares occupied by hidden pieces of the opponent. Keys and values for these tokens are passed into transformer decoder layers. The inputs (**C**) and outputs (**D**) to the decoder are the types and predictions about the types of the opponent hidden pieces, respectively, in row-major order; the inputs use learned positional embeddings.

Stratego is a board wargame played on a 10-by-10 grid with 92 occupiable squares and 2 blocks of non-occupiable squares called lakes (Extended Data Fig. 3a). Each player begins with 40 pieces, arranged in secret on the first 4 rows of their side so that the identities are concealed from the other player. The game proceeds in alternating turns. On each turn, the acting player moves one piece. If the piece is moved onto a square occupied by a piece of the other player, a battle occurs, at which point both pieces are revealed and at least one of them is removed from the board. Victory is achieved by capturing the Flag of the other player or by leaving the other player with no legal moves; the game ends in a draw if the player to move has no legal moves and the other player would have none were it their turn.

#### Piece details

A description of the pieces is provided in Extended Data Fig. 3b. Movable pieces—aside from Scouts—can move one square in a cardinal direction (that is, up, down, left or right) to squares that are either empty or occupied by an opponent piece; Scouts can move any number of squares in a cardinal direction to squares that are either empty or occupied by an opponent piece, so long as the movement does not jump over a lake or an occupied square. When pieces engage in combat, the outcome is typically determined by rank, which means that the higher-ranking piece defeats the lower-ranking piece if their ranks differ and that both are defeated if their ranks are the same.

#### **Additional rules for competitive play**

In competitive play, there are two additional rules. One is the two-square rule, which prohibits a piece from crossing the same square boundary on more than three consecutive turns of its owner. The other is the continuous-chasing rule, which sets limits on the ability of the pieces of one player to chase—in the sense defined below—those of the other.

#### **Threats, evades, chases and chasing**

We define a threat as an action that moves a piece adjacent to a piece of the opponent. An evade is an action that moves a piece that was threatened on the previous turn away from the piece that threatened it. A chase is an unbroken sequence of alternating threats and evades. Finally, chasing is the act of making threats during a chase.

The continuous-chasing rule states that a player who is chasing may not make a threat that would result in a position that has already taken place during the chase, unless that threat would return the moved piece to the square it occupied before the previous turn of the chasing player.

#### **Additional rule for online play**

There is also an additional rule implemented by Strategus, which is both the primary website for online competition and the website on which the evaluation against Pim Niemeijer was conducted. This rule is called the 200-move rule and states that the game ends in a draw if there is a sequence of 200 moves without a battle.

#### Time controls

Competitive Stratego also includes time controls. The evaluation against Pim Niemeijer was conducted under the default 15+3 Strategus time controls. The notation 15+3 means that each player starts with a 15-minute buffer and is allocated 3 free seconds for each move before their buffer starts to run down.

### Engineering details

To make the project feasible on modest academic compute infrastructure, we implemented a graphics processing unit (GPU)-accelerated Stratego simulator in CUDA C++. This reduced the runtime of our data collection, training and search pipelines, while simultaneously reducing memory usage.

Our simulator was designed around five desiderata. First, it avoids explicitly storing in memory quantities that are easily recomputed. For example, we eliminate the need for a traditional rollout buffer that stores large information states and legal action masks by implementing a simulator capable of travelling back in the history of a game and reconstructing quantities of interest on-demand. This greatly reduces memory footprint and fragmentation, as discussed in the ‘Simulator and rollout buffer design’. Second, it maximizes simulation throughput, aiming for roughly 10 million board state updates per second, while also implementing anti-chasing rules. Third, it minimizes dynamic memory allocations: the simulator allocates memory as needed at construction time and then manages that memory directly rather than dynamically allocating and deallocating over time. Fourth, it supports search by enabling fast reset of board states to non-terminal states. Finally, it treats boards independently: once one game terminates, it is reset independently of whether the other games simulated in parallel have terminated. As a consequence, the boards gradually desynchronize, creating a distribution of training data that covers the different phases of the game.

#### Simulator and rollout buffer design

Unlike typical reinforcement learning infrastructure, we do not distinguish between the rollout buffer and the simulator. Rather, we expose a single object, called StrategoRolloutBuffer, that serves both purposes. This object tracks a tunable number *N* of parallel games and is responsible for two critical tasks: supporting historical queries (for example, returning a player’s legal action mask or information-state encoding at a past state); and ‘stepping’ the games by receiving actions and updating their states. The latter is performed by calling the ApplyActions method, which expects a tensor of *N* actions as input.

Once a game terminates, a new game starts. A new game is created by sampling a new initial board for both players. The distribution from which the initial board is sampled can be customized. It is also possible to ask the simulator to reset terminated games to a specific non-initial game state; this feature is important to support search, as discussed in ‘Reset behaviour and support for search’.

The rollout buffer tracks game states in a circular, preallocated GPU-memory buffer of tunable length. Correspondingly, queries about past states can be supported only if the past state is recent enough.

We found that this design, which integrates aspects of a traditional rollout buffer with a simulator, substantially reduces both memory fragmentation and usage compared with implementing a separate rollout buffer (for example, using PyTorch). A separate rollout buffer may need to make copies of legal action masks and information-state tensors, and pack them into aggregate tensors allocated by that buffer. Instead, our StrategoRolloutBuffer is able to reconstruct on-demand past information states and legal action masks. The training loop simply needs to remember at what historical time step the quantity needs to be computed, and then ask the backend to materialize the appropriate tensor to query the value and policy networks. Furthermore, this design is natural when considering that a Stratego simulator needs to track history (at least to a non-trivial extent) to implement the anti-chasing rules described in ‘Rules of Stratego’. Finally, by endowing the simulator with a notion of history, it becomes possible to implement efficient capturing of past states, as needed to efficiently implement search.

#### Two-square rule

To efficiently implement the rule, we built a custom state machine whose state is tracked and updated by StrategoRolloutBuffer.

The state machine is implemented internally by tracking the last four positions occupied by the last-acting piece for each player. The update logic runs directly on the GPU. Special care needs to be taken to properly account for Scouts, which have special movement abilities.

#### Continuous-chasing rule

To properly implement the rule, the simulator requires access to the board history of each game. To quickly detect whether a move would violate the rule, we used a state machine design paired with a fast diffing algorithm. Custom logic was added to make the rule compatible with resetting terminated games to start from non-initial states (as needed to support search; see ‘Reset behaviour and support for search’). Indeed, in this case, the continuous-chasing rule needs to be tested against the history of board states that leads to the non-initial reset state, rather than the history of boards stored in the rollout buffer.

#### Reset behaviour and support for search

To support search, the simulator needs to ‘pin’ the simulation of boards to start from a given state. This ability is implemented in our code by asking the StrategoRolloutBuffer to reset terminated boards not from an initial state, but rather from a custom non-initial state.

Further implementation details—including the simulator application programming interface (API), information-state representation, training configuration, network architectures and search hyperparameters—are provided in Supplementary Information.

### Other experiments

**Extended Data Fig. 5:**
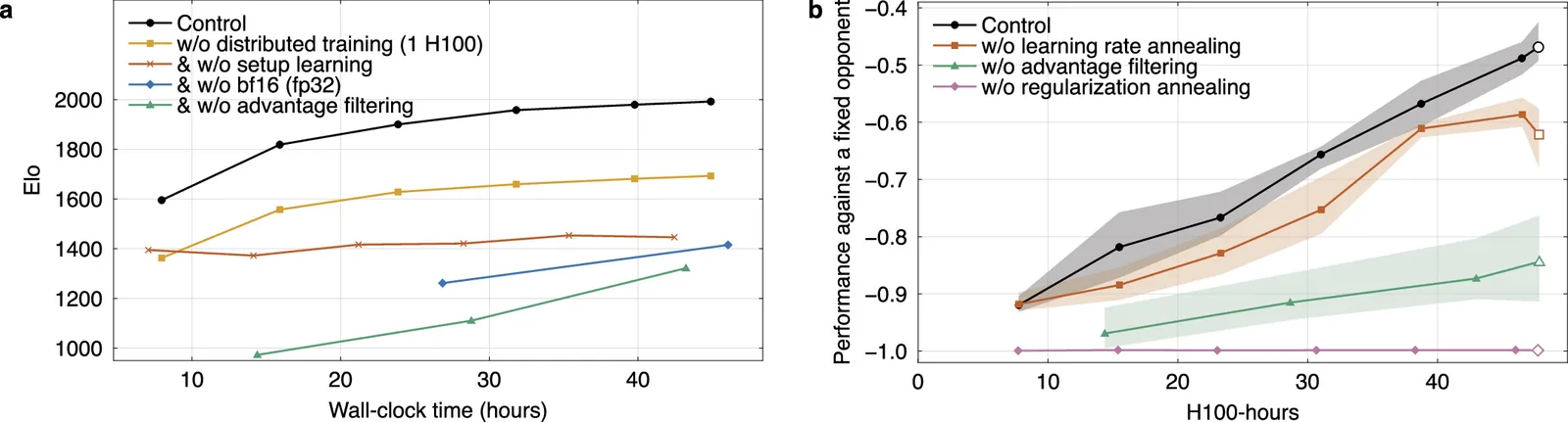
Ablations of the training run. **a** Single-seed ablations. Without distributed training (yellow), Ataraxos follows a similarly shaped trajectory of improvement over the course of scheduled annealing, but at a substantially lower Elo. Series marked with ‘&’ additionally remove the indicated component. When set-up learning is also removed (and replaced by a fixed uniform distribution over set-ups), the shape of the Elo trajectory flattens—both because of the weakness of uniformly distributed set-ups and because of the bad assumptions the associated self-play distribution causes the move network to make about the pieces of its opponents. Removing bfloat16 and advantage filtering substantially slows iterations due to more expensive network calls and larger training datasets, respectively. Removing advantage filtering also causes a large increase in move entropy and large decrease in sample efficiency. **b** Ablations of learning-rate annealing, advantage filtering, and regularization annealing across three seeds. Lines show the mean across seeds and shading shows the minimum and maximum seed performance. All three ablated components materially affect performance over the course of training.

We show reinforcement learning ablations in Extended Data Fig. 5 and the performance of search across varying hyperparameters in Extended Data Table 1. All ablations were performed directly from our final hyperparameter configuration, without retuning the remaining hyperparameters. The results therefore reflect the effect of each ablated component within our final design, rather than the best performance attainable under the ablated design choices.

**Extended Data Fig. 6:**
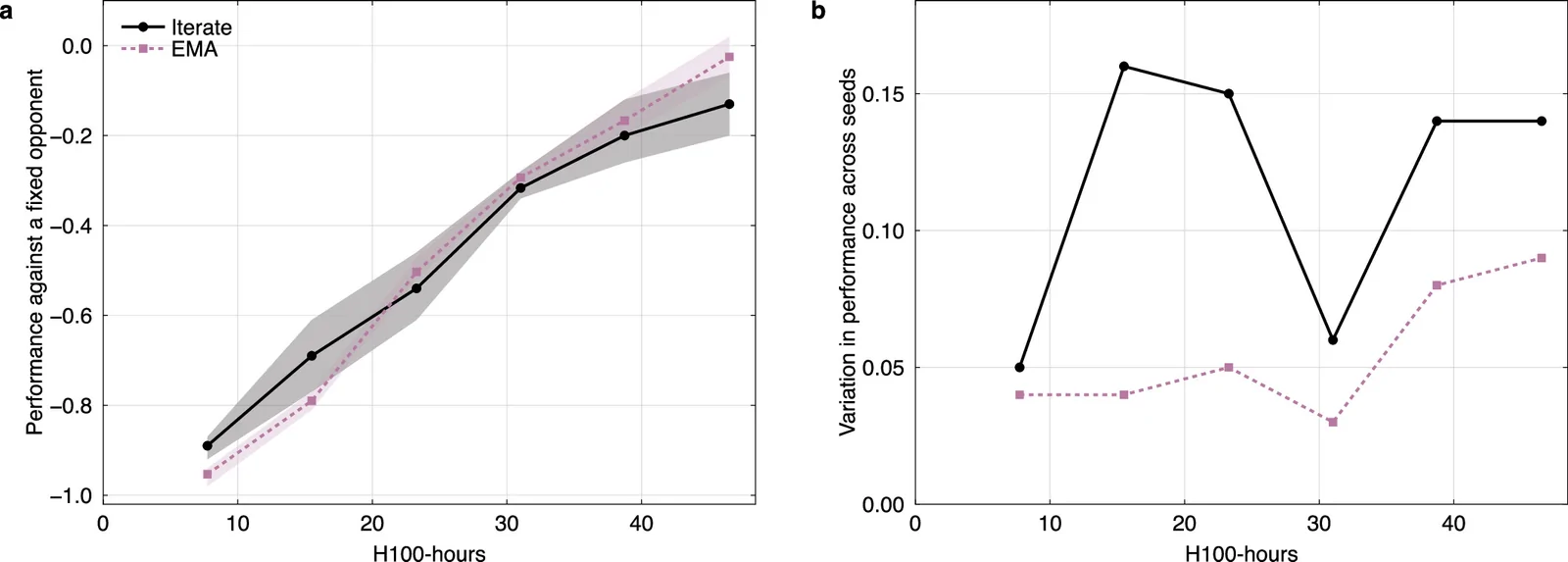
Comparison of current iterates and exponential moving averages across three seeds. **a** Performance against a fixed reference model over training, with the line showing the mean across seeds and the shading showing the minimum and maximum seed performance. **b** Difference at each iteration between the strongest and weakest seed. The exponential moving average attains similar or better mean performance while exhibiting lower variation across seeds.

We additionally compared the performance of the current iterates and their exponential moving averages over training across three seeds by evaluating each against a fixed reference model. As shown in Extended Data Fig. 6, the exponential moving average achieved similar or better mean performance while exhibiting lower variation across seeds.

We also evaluated Ataraxos against the bots from the Stratego Evaluator benchmark^45^: Asmodeus, Celsius, Celsius1.1, Vixen and Peternlewis. Ataraxos won 99, 98, 97, 99 and 98 of 100 games against these opponents, respectively. Win percentages this close to 100 imply that the Stratego Evaluator benchmark is meaningful only as a sanity check.

### Learned set-ups

**Extended Data Fig. 7:**
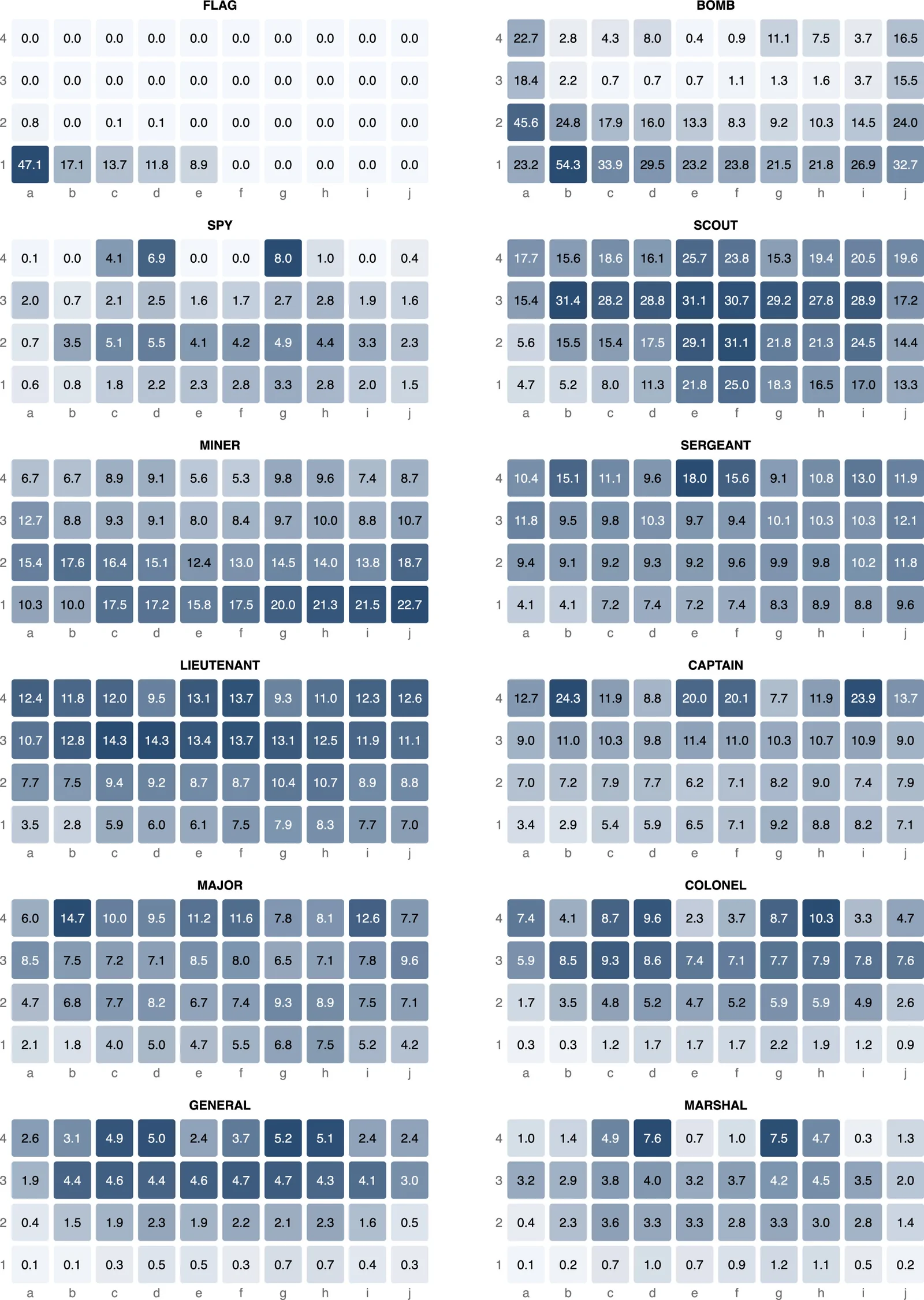
Piece-position frequencies in Ataraxos-generated set-ups. Each value gives an estimated percentage of Ataraxos-generated set-ups in which the indicated piece type occupies that board position, conditional on the Flag being in the left half of the board.

**Extended Data Fig. 8:**
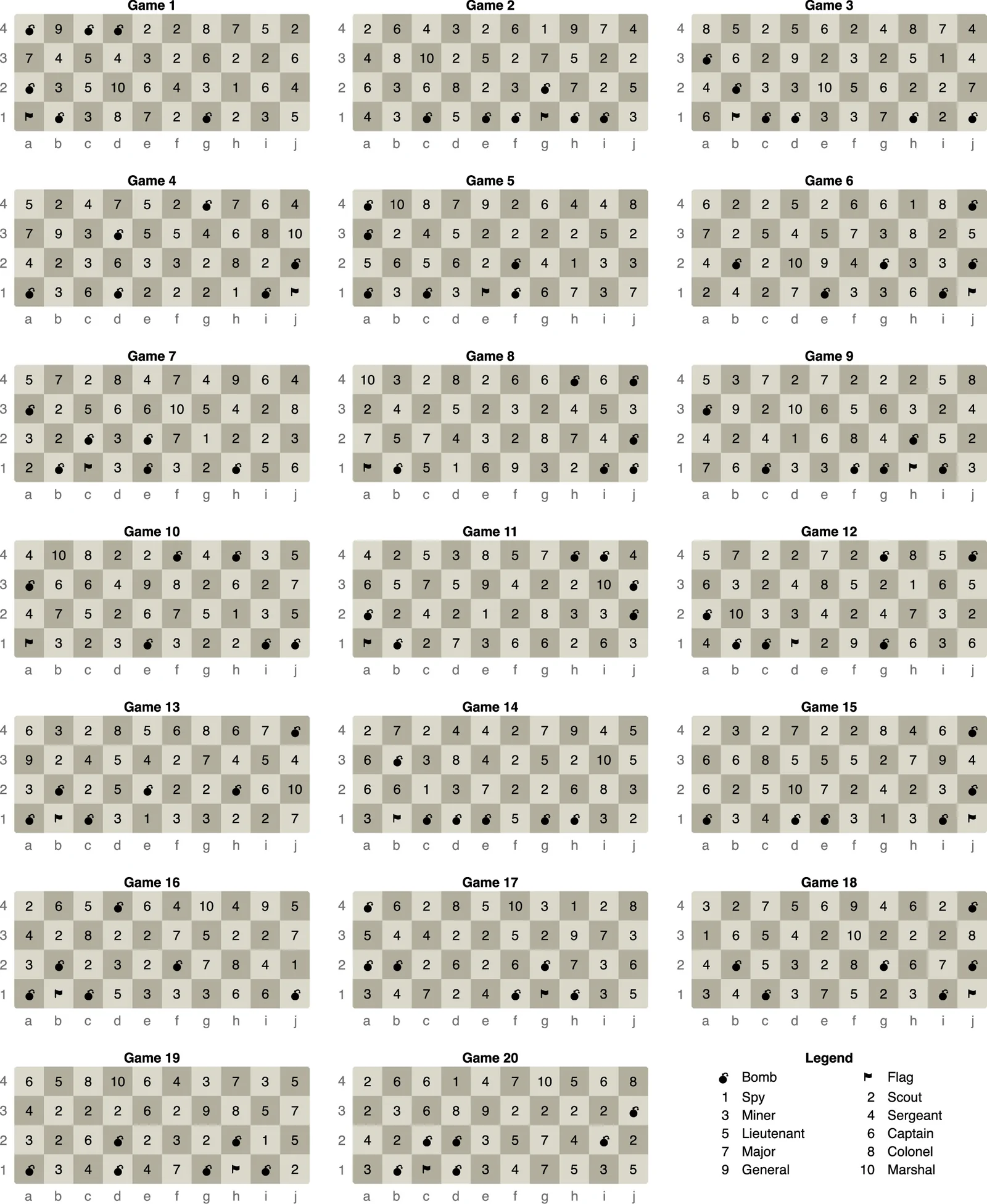
Ataraxos set-ups against Pim Niemeijer. The set-ups Ataraxos played during its 20-game series against Pim Niemeijer, shown in game order.

The probability distribution for each piece in a set-up chosen by Ataraxos is given in Extended Data Fig. 7, assuming that the Flag is on the left side of the set-up. The probabilities for the right side are symmetric, as Ataraxos applies a left–right orientation randomization to its set-ups after sampling from the set-up network to enforce symmetry. Ataraxos uses a bombed-in Flag (that is, a Flag that is enclosed by Bombs, such as in Extended Data Fig. 3a) about two-thirds of the time. The set-ups Ataraxos used in the series against Pim Niemeijer were generated fully autonomously by sampling from this distribution, one per game, and are shown in Extended Data Fig. 8.

### Play style

The play style of Ataraxos differs from those of top human players (which are discussed, for example, by de Boer^46^), in terms of both set-ups and gameplay.

#### Set-ups

Players observed that Ataraxos uses aggressive set-ups (that is, ones in which high-value pieces are at or near the front), high Bombs (that is, Bombs in the third and fourth rows), and back-corner Flags (which humans consider difficult to defend) more frequently than humans. They also observed that it generally uses set-ups that have less predictable structure than those of humans.

#### Gameplay

Players observed the following. Ataraxos moves in a manner that makes its pieces hard to read (that is, discern the types of) relative to the movement patterns of human players. Ataraxos is more willing than humans to accept a draw during the opening when progressing the game would have negative expected value (as happened in Game 11 against Pim Niemeijer). Ataraxos has a stronger preference than humans for preserving Scouts deep into the game. Ataraxos uses certain bluffs sparingly relative to humans, but uses other bluffs that humans consider altogether too risky to play. Ataraxos fights more bitterly than humans when it is behind, aggressively stalling and pestering to slow the progression of its opponent—tactics some humans consider rude. Ataraxos excels relative to humans at long-term positional play, punishing mistakes, defending, playing from an information deficit, transitioning from middle- to end-game, and negotiating favourable positions into wins and unfavourable positions into wins or draws. Ataraxos takes gambles that humans consider arrogant (in the sense of not respecting the opponent). Ataraxos feels preternaturally lucky, always seeming to have the pieces it needs in the right places, to have its gambles pay off and to have its opponents do as it wants.

### Contextualization vis-à-vis DeepNash

DeepNash^1^ is an AI for Stratego that was developed by DeepMind.

#### Evaluation

DeepNash was evaluated on the website Gravon in April 2022, winning 42 of the 50 games counted by Perolat et al.^1^, but not achieving the top ranking on the site. Several aspects of this evaluation are relevant to interpreting its results. (1) By the time of the evaluation, the player base had largely moved away from Gravon (only 25 players are listed for the final ranking of 2022^47^)—as a result, the opponents matched against DeepNash were far from the level of top humans^48^. (2) The one-off online match setting may not have elicited the maximum effort level from the human opponents, who were not aware that an official evaluation was taking place. (3) These opponents had no reason to look for anti-bot exploits, as they were not aware that they were playing against a bot. (4) These opponents were not aware that DeepNash played a fixed strategy (and thus did not know that it was safe to exploit the same weakness across multiple games).

Separately, at the 2023 Stratego World Championship, DeepNash was demoed against human players, recording 19 wins and 9 losses^49^; DeepNash lost to most of the highest-ranked players who played against it, including Pim.

We reached out to DeepMind to ask whether they would allow an evaluation between DeepNash and Ataraxos, offering to build any infrastructure necessary for the evaluation. DeepMind responded that it would not be possible as the code for DeepNash is no longer functional.

#### Compute cost

Perolat et al.^1^ state that DeepNash was trained on 1,024 tensor processing unit nodes. To the recollection of the corresponding author of DeepNash with whom we spoke, this training took between 2 and 3 months and used tensor processing unit v3s. Under 2025 pricing^50^, such a training run would cost roughly between US$3,000,000 and US$4,500,000, depending on how much of the third month was used.

The reinforcement learning models and belief models of Ataraxos were trained on 16 H100s for 1 week and 4 H100s for 4 days, respectively. Such a run costs less than US$8,000 at 2025 prices^51^.

#### Sample cost

The training run for DeepNash consumed about 5.5 billion games and between 5 trillion and 10 trillion training examples.

The training run for Ataraxos consumed about 160 million games and about 50 billion training examples.

### Opportunities for further improvement

#### Learning

Ataraxos accesses history through features rather than learning across time directly. For the belief model, we found that much stronger compute-normalized performance could be attained by interleaving spatial attention with either temporal attention^52^ or recurrent models. However, for reinforcement learning, we did not observe an analogous out-of-the-box compute-normalized performance improvement owing to the additional runtime and memory requirements of such architectures, as well as their interplay with advantage filtering. We believe such architectures could achieve stronger performance given a sufficiently large compute budget or provided with additional runtime, memory or design optimizations.

#### Search

The amount of improvement attainable by the search procedure of Ataraxos is ultimately bounded because it is mimicking a single update step. A more sophisticated search algorithm would be able to leverage arbitrary amounts of additional compute to continue to improve the policy. One possible route towards this end would be to incorporate innovations from knowledge-limited subgame solving^53^.

### Barrage Stratego evaluation details

The evaluations took place on Strategus using the default 5+1 time controls (meaning that each player starts with a 5-minute buffer and is allocated 1 free second for each move before their buffer starts to run down). Games were scheduled by the players at their convenience. Before the start of the evaluation, the players were informed that Ataraxos would not adapt to their play and that they would be evaluated by effective win rate.

Because belief-model training was ongoing at the time at which the evaluation began, we first evaluated the policy network (without search). The policy network won three series: 29 wins, 17 losses and 4 draws (a large margin) against world number-4 Axel Hangg; 36 wins, 12 losses and 2 draws (a very large margin) against world number-3 Sébastien Crot; and 26 wins, 21 losses and 3 draws against world number-1 Pim Niemeijer. We evaluated the search policy in a second 50-game series against Pim, chosen because he had lost to the policy network by the smallest margin. This second series put the search policy at a disadvantage: by the time Pim played it, he had already accumulated information about the set-up policy, which the two policies shared, and about their otherwise similar playstyles. Nonetheless, the search policy substantially outperformed the policy network, winning the series with 31 wins, 14 losses and 5 draws.

Implementation and training details are reported in ‘Barrage Stratego implementation details’ in Supplementary Information.

### Hanabi evaluation details

We evaluated Ataraxos on the two- to five-player variants of Hanabi, with {0, 1, …, *N*} players running search in the *N*-player variant to demonstrate its power as a scalable multi-agent search method. Training, network and search details are reported in ‘Hanabi implementation details’ in Supplementary Information.

**Extended Data Fig. 9:**
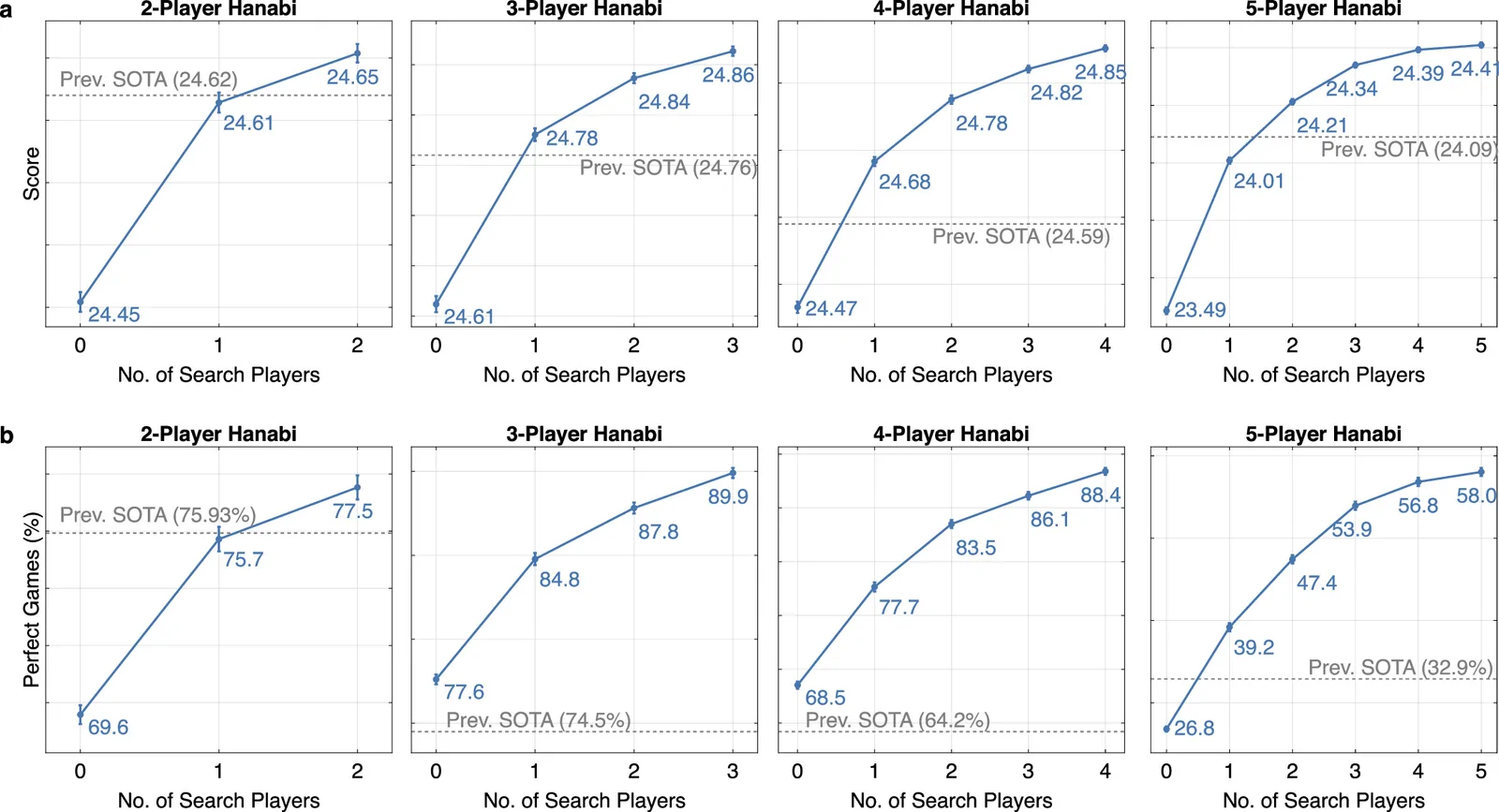
Performance of Ataraxos on Hanabi. Points show means and error bars show standard errors computed over *n* = 10,000 games per condition. **a** Average score. **b** Percentage of perfect (25-point) games.

The evaluation results are summarized in Extended Data Fig. 9, with detailed numbers listed in Supplementary Table 32. All values are mean ± standard error computed over 10,000 games for each condition. With search employed by all players, Ataraxos achieves the following average scores and percentages of perfect (25-point) games:

- 2 players: score 24.654 ± 0.007, perfect games 77.53% ± 0.42%
- 3 players: score 24.863 ± 0.005, perfect games 89.90% ± 0.30%
- 4 players: score 24.852 ± 0.005, perfect games 88.40% ± 0.32%
- 5 players: score 24.410 ± 0.009, perfect games 58.05% ± 0.49%.

In Extended Data Fig. 9a, we plot the performance in terms of average scores over 10,000 games and compare these results with previous state-of-the-art results from ref. ^54^ for the two-player variant and ref. ^55^ for the three-, four-, and five-player variants. Importantly, performance improves monotonically as the number of players using search increases, with particularly large gains for variants with more players as a result of applying Ataraxos search to multiple agents. The gains in the average scores may seem numerically small; however, because Hanabi scores are capped at 25 points and additional points become increasingly difficult to secure near this ceiling, these results reflect substantial progress. Another way to understand this progress is through the percentage of perfect games that reach 25 points. In Extended Data Fig. 9b, we observe large increases in the percentage of perfect games across variants as we apply Ataraxos search to more players.

### *Dou Dizhu* evaluation details

We further evaluated Ataraxos on *dou dizhu*. The reinforcement learning, belief-model and search details are reported in ‘*Dou Dizhu* implementation details’ in Supplementary Information.

We compared Ataraxos against the previous state-of-the-art method, PerfectDou^23^, as well as its predecessor, DouZero^24^. We evaluated heads-up performance in a duplicated setting: one agent plays as the landlord and the opposing agent controls the two independent peasants. Performance is reported as role-averaged score, computed by comparing the same pair of agents after swapping their roles. We evaluated Ataraxos against each baseline over 10,000 duplicated deals and report the performance in Supplementary Table 33. All values are mean ± standard error; comparisons involving Ataraxos use 10,000 duplicated deals, whereas comparisons among the policy network, PerfectDou and DouZero use 100,000 duplicated deals. The role-averaged scores of each agent against the others are as follows:

- Ataraxos: 0.107 ± 0.012 versus the policy network, 0.199 ± 0.015 versus PerfectDou, 0.350 ± 0.016 versus DouZero
- Policy network (i.e., Ataraxos without search):  −0.107 ± 0.012 versus Ataraxos, 0.152 ± 0.004 versus PerfectDou, 0.286 ± 0.004 versus DouZero
- PerfectDou:  −0.199 ± 0.015 versus Ataraxos,  −0.152 ± 0.004 versus the policy network, 0.142 ± 0.004 versus DouZero
- DouZero:  −0.350 ± 0.016 versus Ataraxos,  −0.286 ± 0.004 versus the policy network,  −0.142 ± 0.004 versus PerfectDou.

Ataraxos outperformed existing methods by a large margin and achieved the strongest performance against each baseline, establishing a new state of the art.

As a supplementary analysis, we evaluated Ataraxos under different update step sizes using the role-averaged score against PerfectDou. We report the results in Supplementary Fig. 8. To reduce evaluation cost, this sweep was run with a lighter schedule of 2,500 duplicated deals per setting.

### Ethics statement

The Carnegie Mellon University Institutional Review Board (CMU IRB) determined that the human-player evaluation study qualified for exemption under the 2018 Common Rule, 45 CFR 46.104(d)(3)(i)(B), as a low-risk benign behavioural intervention (STUDY2025_00000013; modification MOD202500000509). Informed consent was obtained from all participants in accordance with the CMU IRB-reviewed consent procedures.

## Online content

Any methods, additional references, Nature Portfolio reporting summaries, source data, extended data, supplementary information, acknowledgements, peer review information; details of author contributions and competing interests; and statements of data and code availability are available at https://doi.org/10.1038/s41586-026-11036-y.

## Supplementary information

Supplementary Information Supplementary notes, tables, results and references.

Peer Review File

## Figures and Tables

**Extended Data Table 1:**
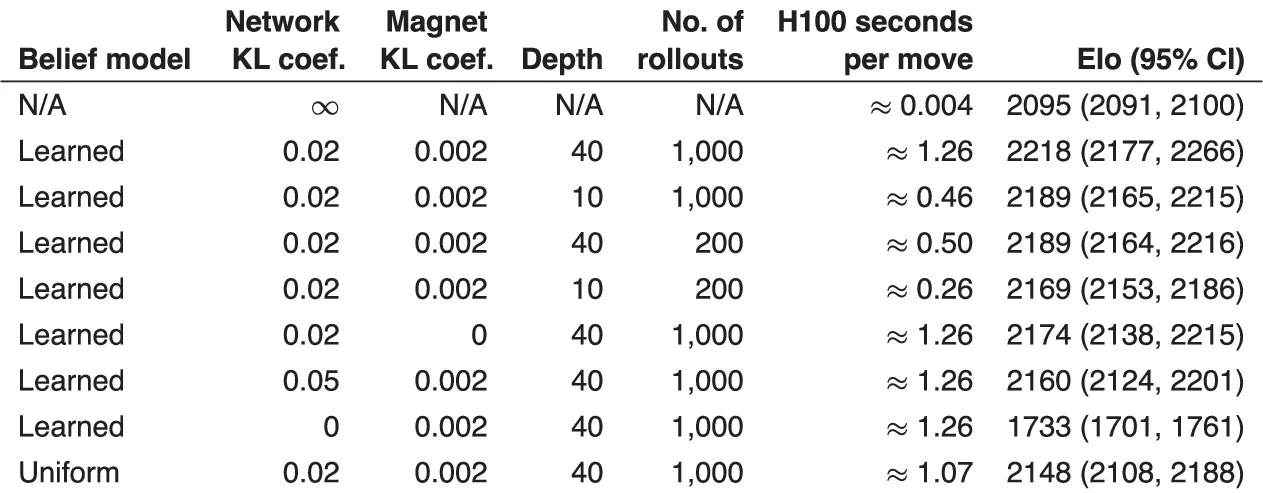
Elo of search with varying hyperparameters

The first row shows direct sampling from the move network (i.e., no search); the second shows the search that was used in the evaluation. Both Elo and time expended increase with the depth of the search and the number of rollouts. Reverse Kullback–Leibler regularization strength has a large effect on performance. Removing the reverse Kullback–Leibler term to the move network devastates performance—causing it to drop well below that of the move network—because the search overfits to idiosyncrasies of the move network that fail to generalize to other opponents. The final row ablates the learned belief model, replacing it with the belief associated with the uniformly random policy.

## Data Availability

No static training datasets were used in this project; all training data were generated from scratch by the agents as they learned. Static records of the 20 evaluation games against Pim Niemeijer are available at https://ataraxosai.github.io.

## Code availability

Code used for the Stratego, Hanabi and *dou dizhu* experiments is publicly available at https://github.com/AtaraxosAI.
