# Supplementary material for: Scalable decision-making for games of imperfect information
Source: Nature. 2026 Sep 30;658(8134):55–9. doi: 10.1038/s41586-026-11036-y (PMC13626944; doi:10.1038/s41586-026-11036-y)
Supplement: Supplementary file 1 [file 41586_2026_11036_MOESM1_ESM.pdf]

nature portfolio

https://doi.org/10.1038/s41586-026-11036-y

Supplementary information

Scalable decision-making for games of imperfect information

In the format provided by the authors and unedited

Supplementary Information

Scalable Decision Making for Games of Imperfect Information

Samuel Sokota, Eugene Vinitsky, Hengyuan Hu, Zhiyuan Fan,
J. Zico Kolter, and Gabriele Farina

The sections below provide detailed implementation, training, and evaluation material supporting the Methods and Extended Data figures and tables.

S1 Simulator API

The following table describes the main historical queries supported by the simulator, complementing the Methods subsection “Simulator and Rollout Buffer Design”.

Table S1: Historical simulator queries exposed by the API.

Method	*Input*	*Output*	*Description*
`CurrentStep`	(none)	64-bit integer	Returns the number of state updates performed by the simulator.
`ActingPlayer`	time step	0 or 1	The player acting at the current time step (0 for the red player, 1 for the blue player).
`ComputeLegalActionMask`	time step	(none)	Computes the legal action mask for the acting player at the given time step. The answer is written in a preallocated tensor kept by the rollout buffer.
`ComputeInfostateTensor`	time step	(none)	Computes the information state for the acting player at the given time step. The answer is written in a preallocated tensor kept by the rollout buffer.
`ComputeRewardPl0`	time step	(none)	Computes the reward for the red player at the given time step. The reward is set to 0 for those boards that are not terminated at the given time step. The answer is written in a preallocated tensor kept by the rollout buffer.
`ComputeIsUnknownPiece`	time step	(none)	Computes a boolean tensor indicating which pieces are unknown to the acting player. The answer is written in a preallocated tensor kept by the rollout buffer.

`ComputePieceTypeOnehot`	time step	(none)	Computes a one-hot encoding of the piece types on the board from the perspective of the acting player. The answer is written in a preallocated tensor kept by the rollout buffer.
`ComputeTwoSquareRuleApplies`	time step	(none)	Computes whether the two-square repetition rule applies at the given time step. The answer is written in a preallocated tensor kept by the rollout buffer.
`GetTerminatedSince`	time step	Tensor	For each game, computes the number of steps since it terminated. For non-terminated games, this value is 0. Returns a non-owning tensor.
`GetHasLegalMovement`	time step	Tensor	Computes whether the acting player has at least one legal move. Returns a non-owning tensor.
`GetFlagCaptured`	time step	Tensor	Computes whether a player has captured the opponent's flag. Returns a non-owning tensor.
`GetPlayedActions`	time step	Tensor	Retrieves the action taken at the specified time step for each game. Returns a non-owning tensor.
`GetNumMoves`	time step	Tensor	Gets the total number of moves played in each game. Returns a non-owning tensor.
`GetNumMovesSinceLastAttack`	time step	Tensor	Gets the number of moves since the last attack for each game. Returns a non-owning tensor.
`BoardStrs`	time step	Vector of strings	Returns a string representation of the board state for each game at the current time step. Useful for debugging.

`SampleRandomLegalAction`	GPU-allocated tensor	(none)	Fills out the provided GPU-allocated tensor with a legal action drawn uniformly for each game at the current step.

S2 Stratego Information State Representation

The representation of the information state for each player comprises two components:
- A representation of the current *board state*; and
- An encoding of the past K moves.

We describe each component in detail in the following subsections.

S2.1 Representation of the Board State

We encode information about the state of the board and the history of the game as a collection of channels, each of which is a 10×10 matrix of floating point numbers. The channels can be divided into different logical subgroups, as follows.

Basic piece properties The following channels expose basic information regarding the pieces on the board, including their visibility and whether they have moved in the past.

Channel(s)	Description
0–11	Indicator of whether the piece occupying each square belongs to the player and has type T, for each of the 12 types $T \in \{$Spy, Scout, Miner, Sergeant, Lieutenant, Captain, Major, Colonel, General, Marshal, Flag, Bomb$\}$.
12–23	For each square, probability that it belongs to the opponent and has type T for each of the 12 types $T \in \{\text{Spy}, \dots, \text{Bomb}\}$, assuming the setup and moves were selected uniformly at random.
24–35	Same as channels 12–23, but from the point of view of the opponent player.
36	Indicator of what squares on the board are occupied by hidden pieces of the player.
37	Indicator of what squares on the board are occupied by hidden pieces of the opponent.
38	Indicator of what squares on the board are empty.
39	Indicator of what squares on the board are occupied by pieces of the player that moved.
40	Indicator of what squares on the board are occupied by pieces of the opponent that moved.

Channel(s)	Description
41	Fraction of the maximum number of moves per game (see Section S3.1) that have been exhausted.
42	Fraction of the maximum number of moves between attacks (see Section S3.1) that have been exhausted.

Threats, evasions, attack opportunities The following channels encode information regarding the historical behaviour of pieces, including whether pieces threatened or evaded other pieces, and whether pieces opted not to attack other pieces when they had the opportunity.

For the purposes of the next channels, we recall that a *threat* is a movement of one's piece so as to make the piece adjacent to a piece of the opponent. The opponent's piece is considered *threatened*.

Channel(s)	Description
43–52	Indicator of whether the piece occupying the square belongs to the player, and has ever threatened a piece of the opponent which, at the time of the threat, was a revealed piece of type $T \in \{\text{Spy}, \dots, \text{Marshal}\}$.
53	Indicator of whether the piece occupying the square belongs to the player, and has ever threatened a piece of the opponent which, at the time of the threat, was hidden.

An *evasion* is realized when a piece that was threatened in the immediately preceding move is moved away from the threatening piece, to an empty square.

Channel(s)	Description
54–63	Indicator of whether the piece occupying the square belongs to the player, and has ever evaded a threat from an opponent's piece which, at the time of the threat, was a revealed piece of type $T \in \{\text{Spy}, \dots, \text{Marshal}\}$.
64	Indicator of whether the piece occupying the square belongs to the player, and has ever evaded a threat from an opponent's piece which, at the time of the threat, was hidden.

Finally, we define a piece to be *actively adjacent* to an opponent's piece when the player controlling the former has had the chance to attack the latter, but opted not to.

Channel(s)	Description
65–74	Indicator of whether the piece occupying the square belongs to the player, and has ever been actively adjacent to an opponent's piece which, at the time of the adjacency, was a revealed piece of type $T \in \{\text{Spy}, \ldots, \text{Marshal}\}$.
75	Indicator of whether the piece occupying the square belongs to the player, and has ever been actively adjacent to an opponent's piece which, at the time of the adjacency, was hidden.

The same information regarding threats, evasions, and active adjacencies is then reported from the point of view of the opponent.

Channel(s)	Description
76–85	Same as channels 43–52, but from the point of view of the opponent player.
86	Same as channel 53, but from the point of view of the opponent player.
87–96	Same as channels 54–63, but from the point of view of the opponent player.
97	Same as channel 64, but from the point of view of the opponent player.
98–107	Same as channels 65–74, but from the point of view of the opponent player.
108	Same as channel 75, but from the point of view of the opponent player.

Captured pieces The next group of channels encodes the starting positions of the pieces that have been captured.

Channel(s)	Description
109–119	Indicator of whether the given square was occupied, at the start of the game, by a piece of the player of type $T \in \{\text{Spy}, \ldots, \text{Marshal}, \text{Bomb}\}$ that has now been captured.
120–130	Indicator of whether the given square was occupied, at the start of the game, by a piece of the opponent of type $T \in \{\text{Spy}, \ldots, \text{Marshal}, \text{Bomb}\}$ that has now been captured.

Causes of death The following channels encode the locations in which pieces died, as well as what interaction caused their death. The following causes of death are distinguished:

1. The piece attacked an opponent piece of strictly stronger rank, which was revealed at the time of the attack.
2. The piece attacked an opponent piece of equal rank, which was revealed at the time of the attack.

3. The piece attacked an unrevealed opponent piece, and lost the battle.
4. The piece was revealed, and was attacked by an opponent piece (revealed or not). It lost the attack because it was strictly weaker.
5. The piece was revealed, and was attacked by an opponent piece (revealed or not). The battle was a tie.
6. The piece was unrevealed, and was attacked by an opponent piece (revealed or not).

**Channel(s)**	**Description**
131–140	Whether a piece of the player of type $T \in \{\text{Spy}, \dots, \text{Marshal}\}$ died in this square, due to cause of death 1.
141–150	Whether a piece of the player of type $T \in \{\text{Spy}, \dots, \text{Marshal}\}$ died in this square, due to cause of death 2.
151–180	Whether a piece of the player of type $T \in \{\text{Spy}, \dots, \text{Marshal}, \text{Bomb}\}$ died in this square, due to causes of death 3–5 (channels 151–160, 161–170, and 171–180, respectively).
181–190	Whether a piece of the player of type $T \in \{\text{Spy}, \dots, \text{Marshal}, \text{Bomb}\}$ died in this square, due to cause of death 6.
191–250	Same as channels 131–190, but for the opponent.

Protection moves The final group of channels refers to protection moves. When a player's piece A threatens a piece B of the opponent, the opponent might respond by moving one of their pieces, say C, to become adjacent to B. This way, if the player follows through with the aggression, the opponent can attack A with C. Moving C to become adjacent to B as a response to a threat from A is a *protection* move. More precisely, we say that piece C protected piece B against piece A; equivalently, piece B was protected by piece C against piece A. We extend the same terminology to the case in which B is an empty square.

**Channel(s)**	**Description**
251–261	Whether the piece in the square belongs to the player, and has ever protected a piece of the player which, at the time of the protection, had revealed type $T \in \{\text{Spy}, \dots, \text{Marshal}, \text{Bomb}\}$.
262	Whether the piece in the square belongs to the player, and has ever protected an empty square.
263	Whether the piece in the square belongs to the player, and has ever protected a piece of the player which, at the time of the protection, was not revealed.

Channel(s)	Description
264–274	Whether the piece in the square belongs to the player, and has ever protected against a piece of the opponent which, at the time of the protection, had revealed type $T \in \{\text{Spy}, \dots, \text{Marshal}, \text{Bomb}\}$.
275	Padding channel (all zeros).
276	Whether the piece in the square belongs to the player, and has ever protected against a piece of the opponent which, at the time of the protection, was not revealed.

Channel(s)	Description
277–287	Whether the piece in the square belongs to the player, and has ever been protected by a piece of the player which, at the time of the protection, had revealed type $T \in \{\text{Spy}, \dots, \text{Marshal}, \text{Bomb}\}$.
288	Padding channel (all zeros).
289	Whether the piece in the square belongs to the player, and has ever been protected by a piece of the player which, at the time of the protection, was not revealed.

Channel(s)	Description
290–300	Whether the piece in the square belongs to the player, and has ever been protected against a piece of the opponent which, at the time of the protection, had revealed type $T \in \{\text{Spy}, \dots, \text{Marshal}, \text{Bomb}\}$.
301	Padding channel (all zeros).
302	Whether the piece in the square belongs to the player, and has ever been protected against a piece of the opponent which, at the time of the protection, was not revealed.

Channel(s)	Description
303–354	Same as channels 251–302, but for the opponent.

Additional information In addition, the network has access to the following information.

Channel(s)	Description
355–454	Starting location of each piece on the board, one-hot encoded. Specifically, channel $355 + k$ encodes in position $(i, j) \in \{1, \ldots, 10\}^2$ whether the piece currently occupying square (i, j) started from square k, according to a row-major ordering of the squares.

S2.2 Representation of Recent Moves

In addition to the board state channels described above, we also include channels that encode the most recent 32 moves. Each move is encoded in a channel, for a total of 32 additional channels. For each move, a 10×10 matrix of floating point numbers encodes the source and destination square of the movement, with the convention that a $+1.0$ is written in the destination square and a -1.0 in the source square. All other squares are filled with zeros.

S3 Stratego Implementation Details

S3.1 Game Rules

There are two main parameterized game settings, detailed below. One is the value of k for the k-move rule, which states that a draw is declared after k consecutive battleless moves. For training, we used a 100-move rule. We found that this outperformed training under a 200-move rule—even though evaluation took place under a 200-move rule. (The representation of the proximity of the rule to triggering is fractional—see channel 42 in Section S2.1; so n moves without a battle was represented as n/100 during training, but n/200 during testing.) This may be because it was effective at discouraging dawdling, thereby increasing the quality of the generated data. The other game setting is the maximum game length before a draw is declared, which was set to $4{,}000$. This was imposed for edge-case safety and is not an actual rule of Stratego. In practice, we found that it virtually never triggered when paired with a 100-move rule.

Table S2: Game rules training hyperparameters.

Hyperparameter	Value
Number of consecutive battleless moves after which a draw is declared	100
Number of moves after which a draw is declared	$4{,}000$

S3.2 Data Collection

For data collection, each GPU simulated $1{,}536$ independent environments in parallel, playing out 202 moves (101 for each player) for each of these environments between training iterations. (Over 16 GPUs, this yielded about 5 million transitions per training iteration.) The initial boards of new games were sampled using a pool of pre-generated setups (sampled from the setup network), which contained $1{,}000$ setups per player per GPU and was regenerated after each training iteration. These hyperparameters are listed below.

Table S3: Data collection hyperparameters.

**Hyperparameter**	**Value**
Number of parallel environments	$1{,}536$ per GPU
Generated setups per player	$1{,}000$ per GPU
Number of moves between training iterations	202

S3.3 Setup Learning

Given a setup prefix (meaning a setup partially filled in under row-major order), the setup network produces three outputs:

1. A categorical estimate of the probabilities of the possible outcomes of the game (win, loss, draw), given the setup prefix.
2. A real-valued estimate of the conditional entropy of the setup, given the setup prefix.
3. A distribution over the next piece placement, given the setup prefix.

We trained these outputs on all setups for games that finished during the previous data collection period. Since most games span multiple reinforcement learning iterations, the training data was slightly off-policy.

Table S4: Setup learning notation.

**Symbol**	**Meaning**
$\bar{\sigma}$	Setup for which a game was completed.
σ	A prefix of $\bar{\sigma}$.
θ_t	Parameters that generated $\bar{\sigma}$.
θ	Current parameters.
$\pi_{\theta_t}(\bar{\sigma} \mid \sigma)$	Probability of $\bar{\sigma}$ given σ under θ_t.
$h_{\theta_t}(\sigma)$	Predicted conditional entropy of setup prefix σ under θ_t.
$h_{\theta}(\sigma)$	Predicted conditional entropy of setup prefix σ under θ.
o	Outcome in $\{$win=1, loss=-1, draw=0$\}$ of the game started from $\bar{\sigma}$.

Symbol	Meaning
$v_\theta(o \mid \sigma)$	Predicted probability of outcome o given σ under θ.
$\mathbb{E}[v_{\theta_t}(\sigma)]$	Predicted expected outcome (with win=1, loss=-1, draw=0) given σ under θ_t.
α	Regularization temperature (coefficient for entropy maximization).
σ^+	Immediate successor to σ on the path to $\bar{\sigma}$.
$\pi_{\theta_t}(\sigma^+ \mid \sigma)$	Probability of σ^+ given σ under θ_t.
$\pi_\theta(\sigma^+ \mid \sigma)$	Probability of σ^+ given σ under θ.
$\pi_{\theta_t}(\sigma)$	Distribution over next piece type given σ under θ_t.
$\pi_\theta(\sigma)$	Distribution over next piece type given σ under θ.

With the notation above, we used the conditional entropy prediction loss

$$\mathcal{L}_h = \left(\frac{-\log \pi_{\theta_t}(\bar{\sigma} \mid \sigma)}{10} - h_\theta(\sigma) \right)^2; \tag{1}$$

the value prediction loss

$$\mathcal{L}_v = -\log v_\theta(o \mid \sigma); \tag{2}$$

the estimated advantage

$$\delta = (o - \mathbb{E}[v_{\theta_t}(\sigma)]) + \alpha(-\log \pi_{\theta_t}(\bar{\sigma} \mid \sigma) - 10 h_{\theta_t}(\sigma));$$

and the policy loss

$$\mathcal{L}_\pi = -\min\left(r\delta, \text{clip}(r, 0.8, 1.2)\delta\right) + 0.1\text{KL}(\pi_\theta(\sigma), \pi_{\theta_t}(\sigma)), \tag{3}$$

where

$$r = \frac{\pi_\theta(\sigma^+ \mid \sigma)}{\pi_{\theta_t}(\sigma^+ \mid \sigma)}.$$

We weighted these losses as shown below:

$$\mathcal{L}_{\text{setup}} = \mathcal{L}_\pi + \frac{1}{2}\mathcal{L}_v + \mathcal{L}_h. \tag{4}$$

We clipped the gradient norm for this loss at 0.5. We performed 5 epochs over the setups associated with games that finished during the previous data collection period in batches of $1{,}024$, using Adam with a learning rate of 5×10^{-5}. We updated an exponential moving average of the parameters—which was used for evaluation—after completion of the training iteration with a smoothing parameter of 0.999.

These hyperparameter choices are summarized below.

Table S5: Setup learning hyperparameters.

**Hyperparameter**	**Value**
Adam learning rate	5×10^{-5}
Batch size	1,024 per GPU
Number of epochs per training iteration	5
Importance ratio clipping parameter	0.2
Conditional entropy prediction loss coefficient	1
Reverse KL to data collection policy loss coefficient	0.1
Value loss coefficient	0.5
Regularization temperature	$\frac{0.1}{(\text{Training iteration number})^{0.3}}$
Maximum gradient norm	0.5
Normalizing constant for conditional entropy prediction	$1/10$
Exponential moving average smoothing factor	0.999

S3.4 Move Learning

Given a position, the move network produces two outputs:

1. A categorical estimate of the probabilities of the possible outcomes of the game (win, loss, draw), given the position.
2. A distribution over moves that are legal given the position.

We trained these outputs on some but not all of the moves made over the last 202 simulator steps. Specifically, we included a move in the training data if both of the following were true:

1. The magnitude of the estimated advantage was at or above the 0.75 quantile across the data for which advantages were estimated.
2. The magnitude of the estimated advantage was 0.01 or above.

Table S6: Move learning notation.

**Symbol**	**Meaning**
x	A position of the acting player, represented as described in Section S2.
$\{x'\}$	The sequence of positions subsequent to x for the same player.
θ_t	The parameters that played the move at position x.

Symbol	Meaning
θ	The current parameters.
$v_{\theta_t}(x)$	Predicted win, loss, draw probabilities at x under θ_t.
$v_\theta(x)$	Predicted win, loss, draw probabilities at x under θ.
$\mathbb{E}[v_{\theta_t}(x)]$	Predicted expected outcome (with win=1, loss=-1, draw=0) at x under θ_t.
o	The outcome of the game in which x occurred (if game has finished).
δ	λ-return-estimated advantage for move played at x.
ξ	λ-return-estimated probability of winning, losing, and drawing from x.
m	The move that the acting player played at x.
$\pi_{\theta_t}(m \mid x)$	The probability of m at x under θ_t.
$\pi_\theta(m \mid x)$	The probability of m at x under θ.
ρ	Magnet policy that selects piece to move uniformly and moves it uniformly.
α	Reverse KL to magnet policy ρ loss coefficient.
$\pi_\theta(x)$	Probability of each move at x under θ.
$\pi_{\theta_t}(x)$	Probability of each move at x under θ_t.
$\rho(x)$	Probability of each move at x under ρ.

Using the notation above, we estimated the advantage δ using the λ-return with $\lambda = 0.5$ over the values $\{\mathbb{E}[v_{\theta_t}(x')]\}$ (and o if the game finished) and baseline $\mathbb{E}[v_{\theta_t}(x)]$. We estimated outcome probabilities ξ using the λ-return with $\lambda = 0.8$ over the vector values $\{v_{\theta_t}(x')\}$ (and o, as a one-hot vector, if the game finished).

Given these estimates, we updated the move network using the value loss

$$\mathcal{L}_v = \text{cross-entropy}(\xi, v_\theta(x)), \tag{5}$$

and policy loss

$$\mathcal{L}_\pi = -\min\left(r\delta, \text{clip}(r, 0.8, 1.2)\delta\right) + 0.1\text{KL}(\pi_\theta(x), \pi_{\theta_t}(x)) + \alpha\text{KL}(\pi_\theta(x), \rho(x)), \tag{6}$$

where

$$r = \frac{\pi_\theta(m \mid x)}{\pi_{\theta_t}(m \mid x)}.$$

We weighted these losses evenly, as shown below:

$$\mathcal{L}_{\text{move}} = \mathcal{L}_\pi + \mathcal{L}_v.$$

We trained on this loss in 202 batches of positions grouped by simulator step. If not for filtering by advantage magnitude, the size of these batches would have been $1{,}536$ per GPU; with advantage filtering, they were 1/4 or less of that size. We updated the network using Adam with

the learning rate schedule specified below and a maximum gradient norm of 0.267. We performed only one epoch over this training data. Thereafter, we updated an exponential moving average of the parameters—which was used for evaluation—with a smoothing parameter of 0.999.

Table S7: Move learning hyperparameters.

**Hyperparameter**	**Value**
Importance ratio clipping parameter	0.2
Advantage filtering quantile threshold	0.75
Advantage filtering magnitude threshold	0.01
Exponential moving average smoothing factor	0.999
Advantage estimation λ	0.5
Outcome estimation λ	0.8
Reverse KL to data collection policy loss coefficient	0.1
Adam learning rate	$\text{clip}\left(\frac{0.5}{(\text{Training iteration number})^{1.1}}, 5 \times 10^{-6}, 1 \times 10^{-4}\right)$
Reverse KL to magnet policy loss coefficient	$\frac{0.05}{(\text{Training iteration number})^{0.3}}$
Maximum gradient norm	0.267
Number of epochs per training iteration	1
Value loss coefficient	1
Batch size before filtering	$1{,}536$ per GPU

S3.5 Belief Learning

Given a position, the belief network produces a distribution over configurations of opponent hidden pieces. We trained the belief network on every position from a stream of self-play games of the final setup and move networks, with no filtering or subsampling. At each position, the input was the associated information state—represented as described in Section S2—and the target was the ground-truth types of the opponent hidden pieces. Training used teacher forcing to minimize the negative log likelihood of these true opponent hidden piece types.

The associated training hyperparameters are summarized below.

Table S8: Belief learning hyperparameters.

Hyperparameter	Value
Adam learning rate	5×10^{-5}
Maximum gradient norm	0.5
Batch size	game length/2
Data type	bfloat16
Exponential moving average smoothing factor	0.99
Number of environments	1,024

S3.6 Networks

Details about the networks are given below. Pre-layernorm (i.e., layernorm [56] before the sublayer rather than after the residual [57]) was used for all of the networks. The belief architecture is detailed in Extended Data Fig. 4.

Table S9: Setup network information.

Hyperparameter	Value
Depth	4
Embedding dimension	512
Number of heads	8
Learned positional embedding initialization standard deviation	0.1
Feedforward dimension	2,048
Total parameters	12.6 million

Table S10: Move network information.

Hyperparameter	Value
Depth	8
Embedding dimension	384
Number of heads	8
Learned positional embedding initialization standard deviation	0.1
Feedforward dimension	1,536
Total parameters	14.7 million

Table S11: Belief network information.

**Hyperparameter**	**Value**
Encoder depth	6
Number of decoder blocks	4
Number of heads	8
Embedding dimension	512
Dropout	0.2
Learned positional embedding initialization	Kaiming uniform [58]
Feedforward dimension	2,048
Total parameters	57.1 million

S3.7 Search

The search proceeds in the following steps.

1. The search samples roughly
$$\frac{1{,}000}{\text{number of legal actions}}$$
possible configurations of the types of the hidden pieces of the opponent from the belief network, given the position x.
2. The search performs $1{,}000$ rollouts of depth 40. There is at least one rollout for each combination of legal move and sampled opponent configuration. After the initial move, which is forced, the subsequent 39 moves are played according to the move network.
3. The search computes average values $\hat{q}$ for each legal move according to the value output of the move network at the positions reached by the rollouts starting from that move.
4. The search plays
$$M \sim \pi_{\text{search}}$$
where
$$\pi_{\text{search}} = \arg\max_{\pi} \langle \hat{q}, \pi \rangle - \alpha \mathrm{KL}(\pi, \rho) - \beta \mathrm{KL}(\pi, \pi_\theta) \quad (7)$$
$$\propto \left[e^{\hat{q}} \rho^{\alpha} \pi_\theta^{\beta} \right]^{\frac{1}{\alpha+\beta}} \quad (8)$$
and where $\rho = \rho(x)$ is the magnet policy distribution over moves at position x, $\pi_\theta = \pi_\theta(x)$ is the move network distribution over moves at position x, α is the reverse KL coefficient to the magnet policy, and β is the reverse KL coefficient to the move network policy.

The hyperparameters for this procedure are summarized below.

Table S12: Search hyperparameters.

Hyperparameter	Value
Reverse KL to magnet policy loss coefficient	0.002
Reverse KL to policy network loss coefficient	0.02
Number of rollouts	$1{,}000$
Rollout depth	40

S4 Stratego Training Logs

We show loss values and other quantities for the setup network over the training run in Figure S1 and Figure S2. We show loss values and other quantities for the move network over the training run in Figure S3 and Figure S4. For both networks, dynamic damping guides the policy from taking large steps in a heavily regularized regime at the start of training to taking small steps in a weakly regularized regime by the end of training. For the setup network, this can be observed by the simultaneous reduction of 1) the entropy of the network and 2) both the KL divergence relative to the network that generated the setup and the importance ratio clip rate. For the move network, this can be observed by the simultaneous 1) increase in KL divergence relative to the magnet policy and decrease in entropy and 2) decrease in both the KL divergence relative to the policy that made the move and the importance ratio clip rate.

Figure S5 shows other information about the training process and games, including training times, game outcomes, game lengths, and move priority significance.

To assess training progress more directly, we performed pairwise evaluations of checkpoints from the training run whose iteration indices were divisible by $2{,}000$, playing $2{,}000$ games per raw checkpoint pair. The $32{,}000$-iteration checkpoint occurred in two overlapping checkpoint exports, so we averaged its two evaluation blocks into one displayed row and column; entries involving that checkpoint therefore aggregate $4{,}000$ games, whereas the other off-diagonal entries use $2{,}000$ games. Each entry of the resulting 22-checkpoint score matrix gives the average score of the row checkpoint against the column checkpoint, with wins scored as $+1$, draws as 0, and losses as -1. As shown in Figure S6, later checkpoints generally outperform earlier ones, consistent with approximately monotonic improvement over the course of training.

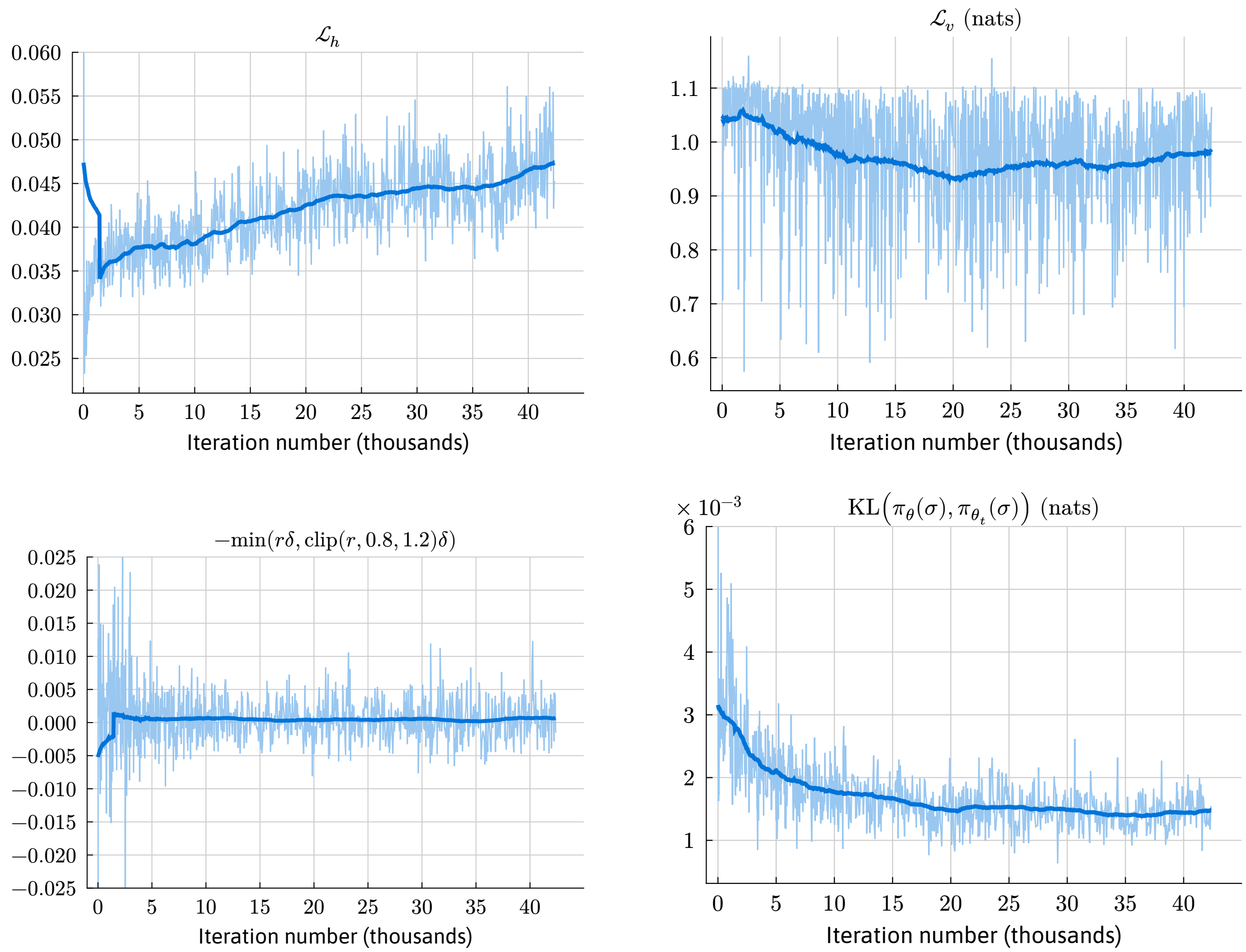

Figure S1: Loss values for setup learning over the training run.

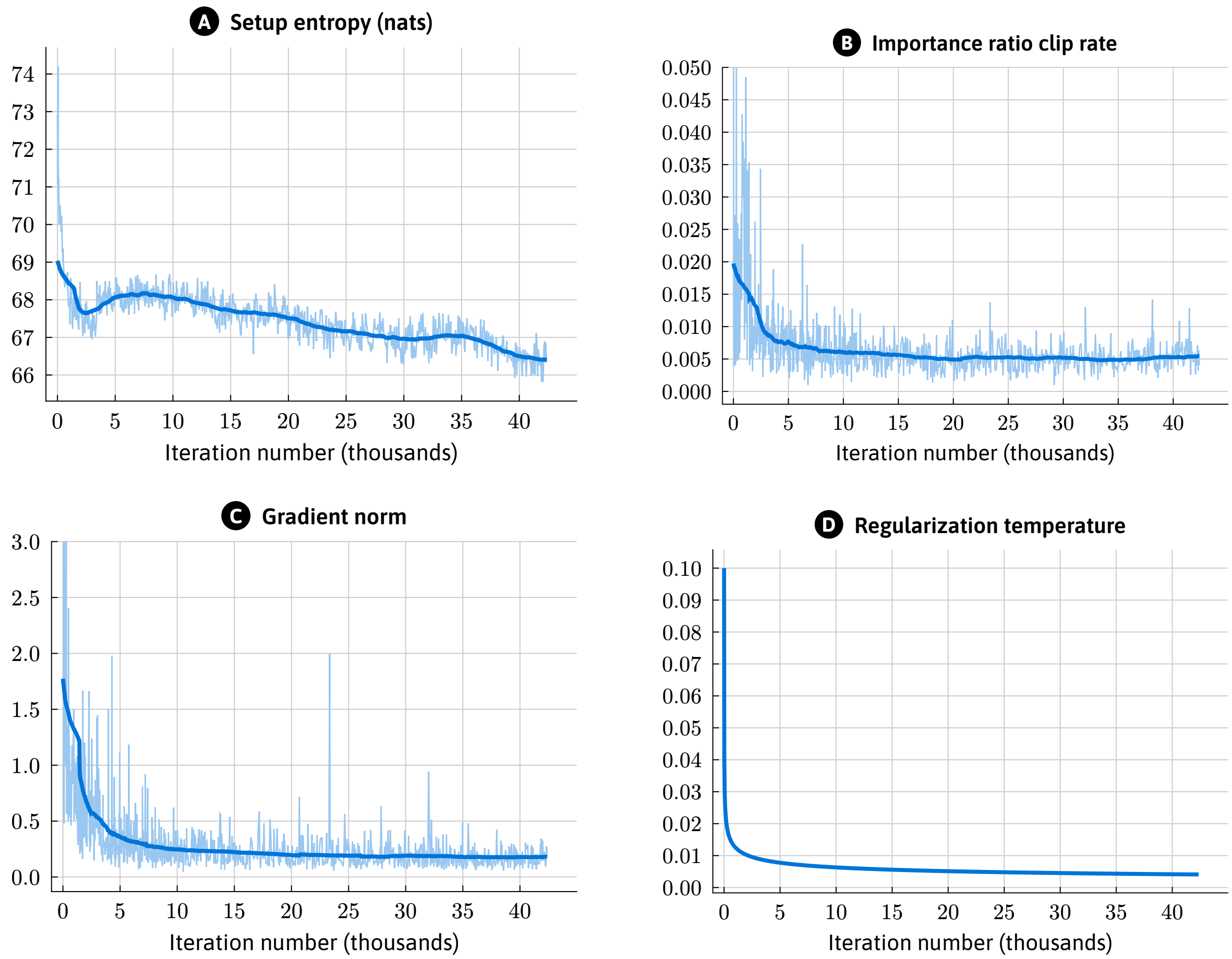

Figure S2: Other setup-related values over the training run. **a** Entropy of the setup distribution. **b** Proportion of data whose importance ratio is clipped. **c** Norm of the gradient of the loss. **d** Entropy maximization coefficient α.

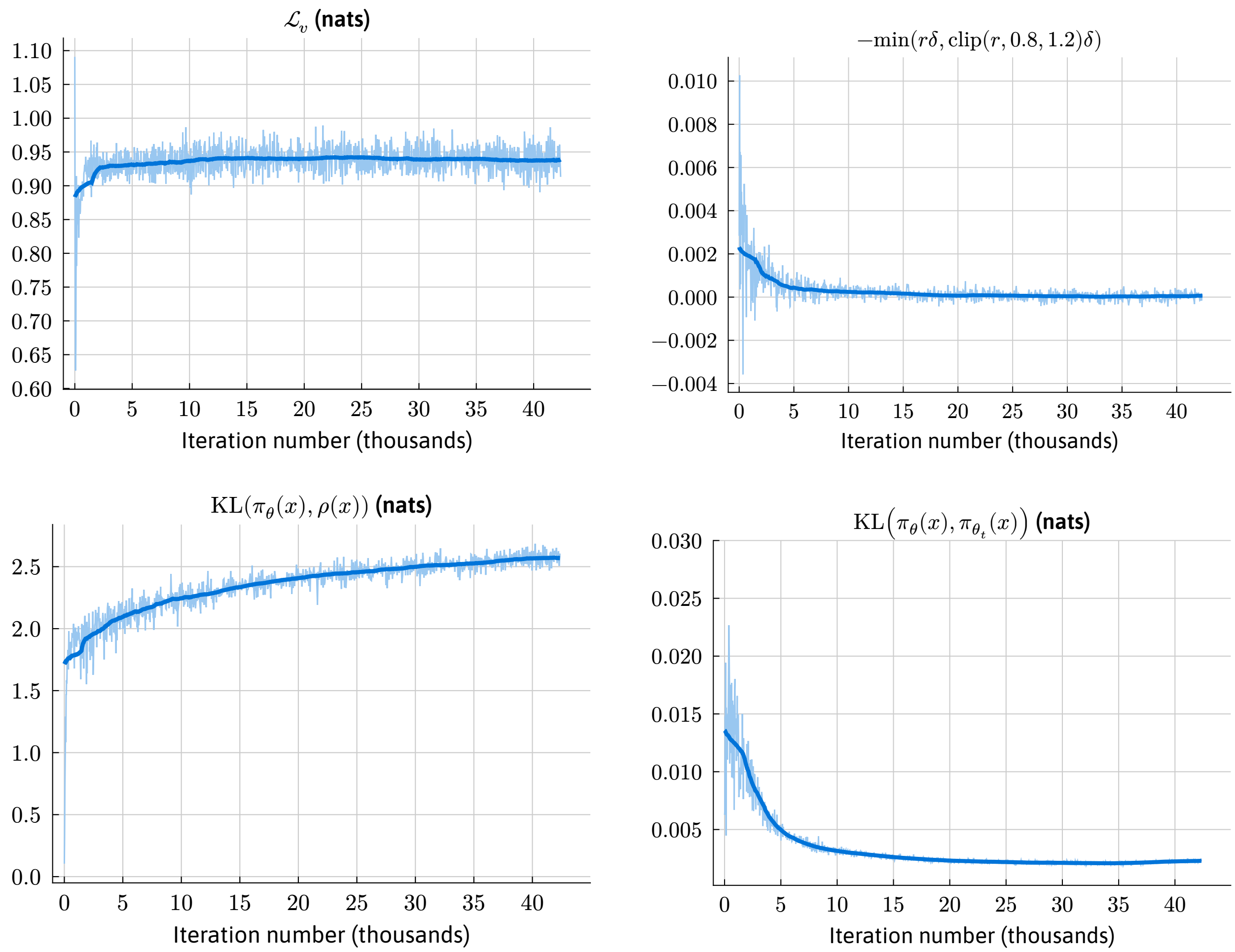

Figure S3: Loss values for move learning over the training run.

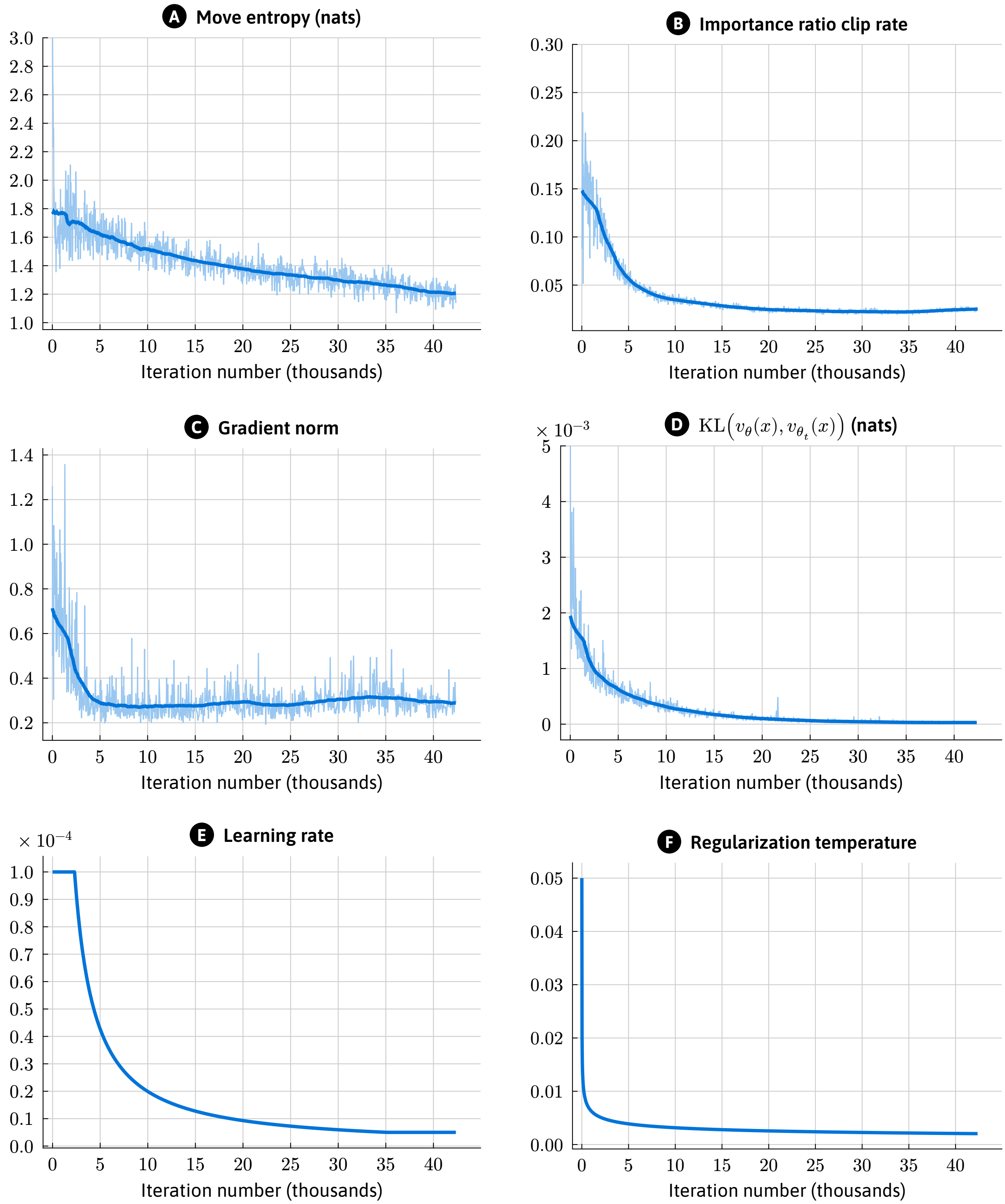

Figure S4: Other move-related values over the training run. **a** Entropy of moves over self-play distribution. **b** Proportion of data whose importance ratio is clipped. **c** Norm of the gradient of the loss. **d** Value function change. **e** Adam learning rate. **f** Coefficient to magnet policy α.

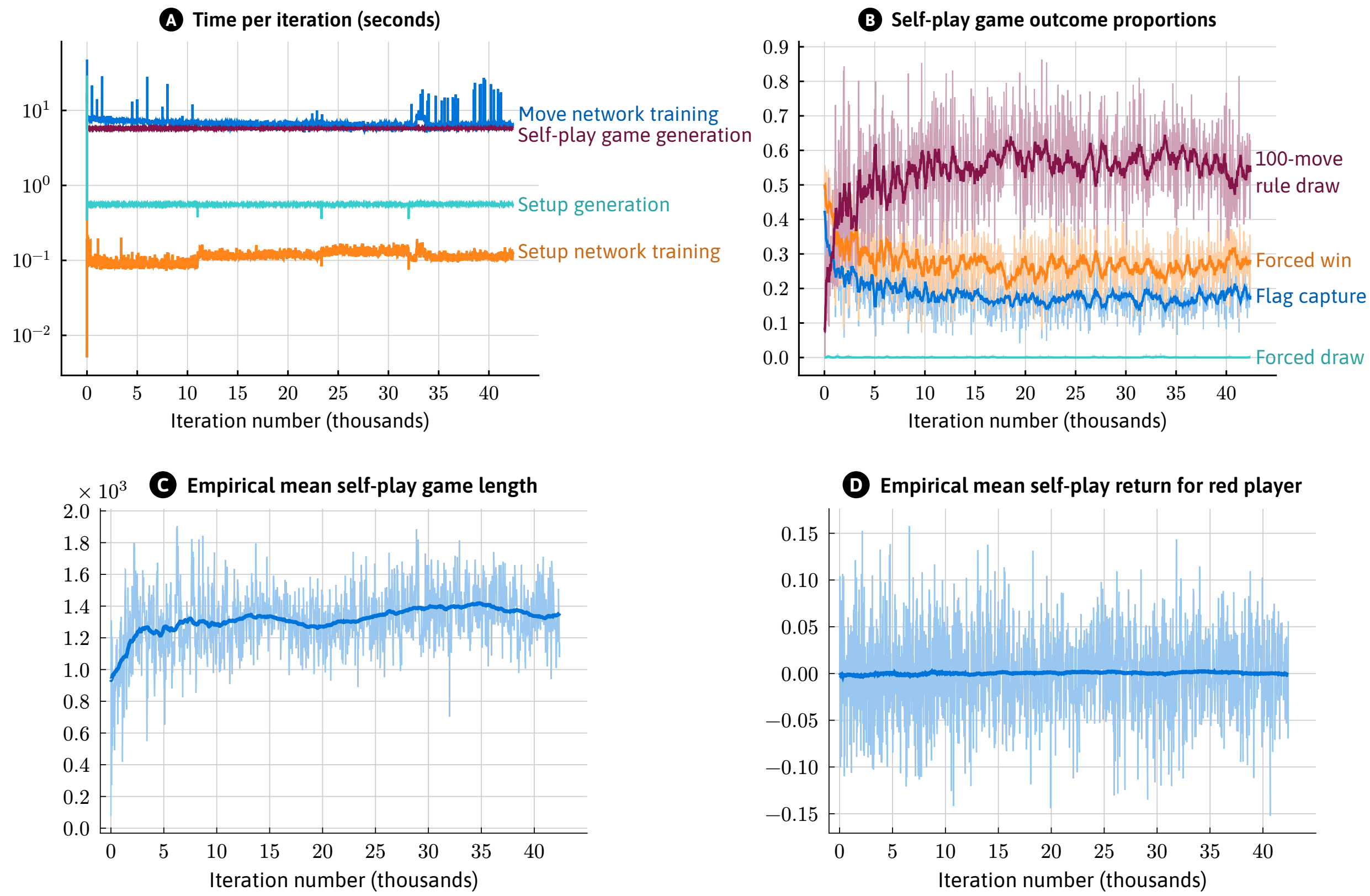

Figure S5: Other information about the training process and games. **a** Time expenditure of different components of the reinforcement learning loop. Iteration speed is bottlenecked by training and data collection for the move network. **b** Proportion over training of each of the four possible game endings: Flag capture, forced win (i.e., by virtue of the other player having no legal moves), forced draw (i.e., by virtue of neither player having legal moves), and a 100-move rule draw (i.e., by virtue of there having been 100 consecutive battleless moves). Ataraxos playing against itself draws a much higher proportion of games than humans playing against one another. **c** The average length of self-play games over training. Ataraxos playing against itself results in much longer games than humans playing against one another. **d** The average return (win=1, loss=-1, draw=0) for the red player (the first moving player) over training. The roughly equal returns of the red and blue players in the self-play of Ataraxos games support the human belief that move priority is not strategically significant in Stratego.

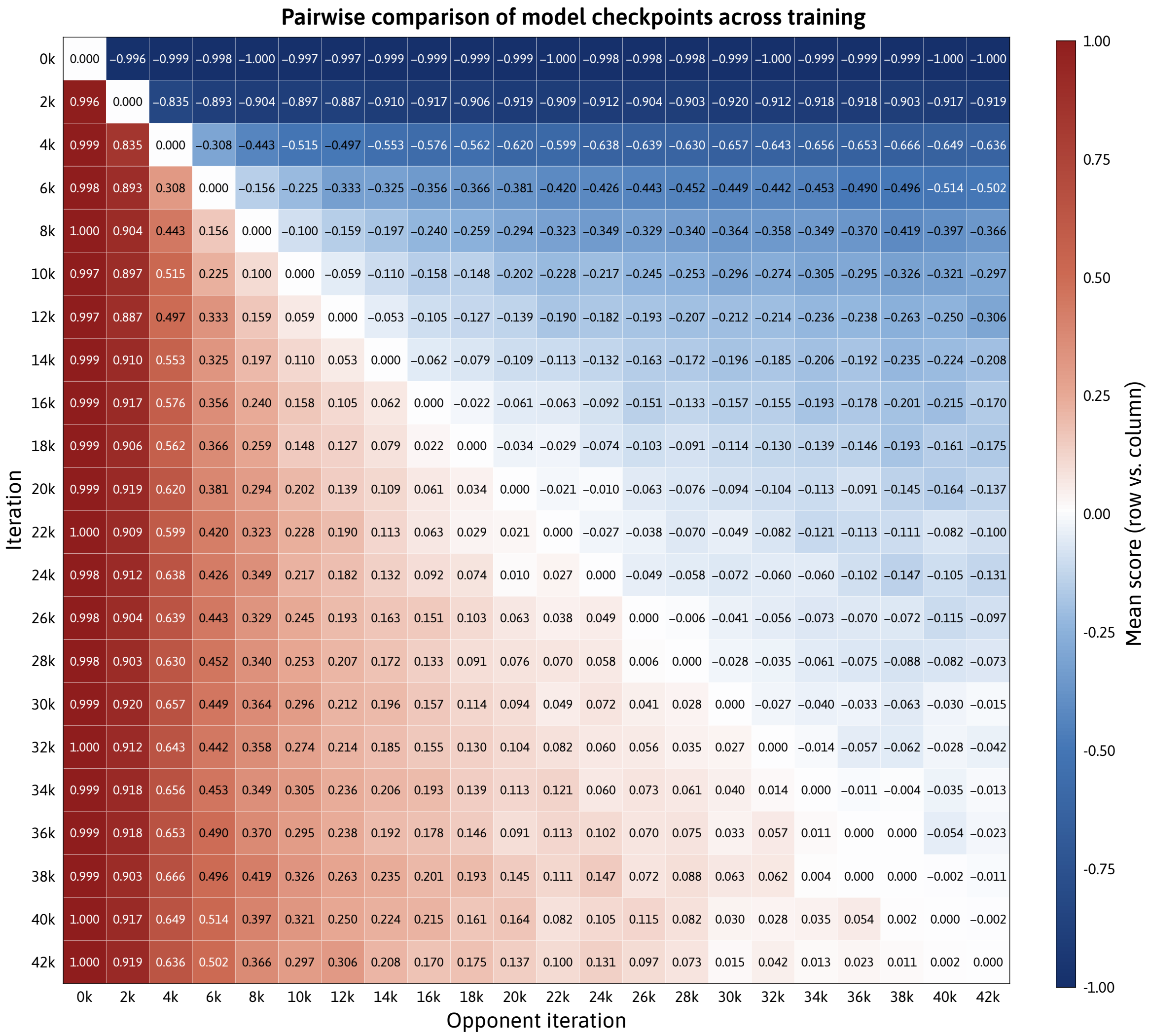

Figure S6: Pairwise evaluation of 22 checkpoints from the training run (those whose iteration indices were divisible by $2{,}000$). Raw checkpoint pairs were evaluated over $2{,}000$ games. The $32{,}000$-iteration row and column average two evaluation blocks and therefore aggregate $4{,}000$ games per off-diagonal entry; all other off-diagonal entries use $2{,}000$ games. Each entry shows the average score of the row checkpoint against the column checkpoint, with wins scored as $+1$, draws as 0, and losses as -1.

S5 Barrage Stratego Implementation Details

Our approach to Barrage Stratego followed the same design as our approach to classic Stratego, with hyperparameters as specified below. Some notable differences from the classic-Stratego configuration were a smaller move network, λ values of zero for advantage and outcome estimation, a constant rather than annealed learning rate for move learning, and a shallower search. The policy network was trained on 4 H100s over 12 days. The belief network was trained on 1 H100 over 2 days.

S5.1 Game Rules

Table S13: Barrage Stratego game rules training hyperparameters.

**Hyperparameter**	**Value**
Number of consecutive battleless moves after which a draw is declared	100
Number of moves after which a draw is declared	2,000

S5.2 Data Collection

Table S14: Barrage Stratego data collection hyperparameters.

**Hyperparameter**	**Value**
Number of parallel environments	1,024 per GPU
Generated setups per player	1,000 per GPU
Number of moves between training iterations	202

S5.3 Setup Learning

Table S15: Barrage Stratego setup learning hyperparameters.

**Hyperparameter**	**Value**
Adam learning rate	5×10^{-5}
Batch size	1,024 per GPU

Hyperparameter	Value
Number of epochs per training iteration	2
Importance ratio clipping parameter	0.2
Conditional entropy prediction loss coefficient	1
Reverse KL to data collection policy loss coefficient	0.1
Value loss coefficient	0.5
Regularization temperature	$\text{clip}\left(\frac{0.2}{(\text{Training iteration number})^{0.2}},\ 0.01,\ 1.0\right)$
Maximum gradient norm	0.5
Normalizing constant for conditional entropy prediction	$1/10$
Exponential moving average smoothing factor	0.999

S5.4 Move Learning

Table S16: Barrage Stratego move learning hyperparameters.

Hyperparameter	Value
Importance ratio clipping parameter	0.2
Advantage filtering quantile threshold	0.5
Advantage filtering magnitude threshold	0.01
Exponential moving average smoothing factor	0.999
Advantage estimation λ	0.0
Outcome estimation λ	0.0
Reverse KL to data collection policy loss coefficient	0.001
Adam learning rate	2.15×10^{-5}
Reverse KL to magnet policy loss coefficient	$\text{clip}\left(\frac{0.04}{(\text{Training iteration number})^{0.3}},\ 0.006,\ 0.1\right)$
Maximum gradient norm	0.4

Hyperparameter	Value
Number of epochs per training iteration	2
Value loss coefficient	0.5
Batch size before filtering	1,024 per GPU

S5.5 Belief Learning

Table S17: Barrage Stratego belief learning hyperparameters.

Hyperparameter	Value
Adam learning rate	2×10^{-5}
Maximum gradient norm	0.5
Exponential moving average smoothing factor	0.99
Number of environments	1,024

S5.6 Networks

Table S18: Barrage Stratego setup network information.

Hyperparameter	Value
Depth	4
Embedding dimension	512
Number of heads	8
Learned positional embedding initialization standard deviation	0.1
Feedforward dimension	2,048
Total parameters	12.6 million

Table S19: Barrage Stratego move network information.

Hyperparameter	Value
Depth	7
Embedding dimension	288
Number of heads	8

Hyperparameter	Value
Learned positional embedding initialization standard deviation	0.1
Feedforward dimension	864
Total parameters	6.1 million

Table S20: Barrage Stratego belief network information.

Hyperparameter	Value
Encoder depth	6
Decoder depth	6
Number of heads	8
Embedding dimension	512
Feedforward dimension	2,048
Dropout	0.1
Total parameters	56.9 million

S5.7 Search

The search procedure is analogous to that of classic Stratego, with the update parameterized by a step size and temperature rather than by two KL coefficients.

Table S21: Barrage Stratego search hyperparameters.

Hyperparameter	Value
Number of rollouts	1,024
Rollout depth	10
Update step size	10
Magnet policy regularization (step size $\times$ temperature)	0.01
Minimum samples per legal action	10

S6 Hanabi Implementation Details

The policy networks were each trained on a single L40 over 2 days. The belief networks were each trained on a single L40 over 1 day.

S6.1 Actor Learning

We trained the base policy using dynamically damped self-play reinforcement learning, with the annealing schedules and other hyperparameters listed below.

Table S22: Hanabi actor learning hyperparameters (shared across 2–5 players).

**Hyperparameter**	**Value**
Importance ratio clipping parameter	0.2
Exponential moving average smoothing factor	0.9999
Advantage estimation λ	0.83
Discount factor γ	0.999
Adam learning rate	$3.5 \times 10^{-4} \left(1 - \left(\frac{t}{T}\right)^{0.4}\right)$
Entropy coefficient	$0.035 \left(1.05 - 0.2 \left(\frac{t}{T}\right)^{2}\right)$
Schedule horizon T	10^7
Maximum gradient norm	0.5
Value loss coefficient	0.5
Batch size	256
Number of parallel environments	$2{,}048$

S6.2 Belief Learning

For belief training, we followed the practice and implementation from prior work [59], with the hyperparameters listed below.

Table S23: Hanabi belief learning hyperparameters.

**Hyperparameter**	**Value**
Adam learning rate	1×10^{-4}
Batch size	128
Number of training steps	10^6
Number of parallel environments	$4{,}800$

S6.3 Networks

We used the exact policy and belief network architectures from prior work [59], which are summarized below.

Table S24: Hanabi actor network architecture.

**Hyperparameter**	**Value**
Private net	3-layer MLP (hidden dim 512, ReLU)
Public net	1-layer MLP (hidden dim 512, ReLU)
Public LSTM	2-layer LSTM (hidden dim 512)
Policy head	Linear ($512 \to |\mathcal{A}|$)
Value head	Linear ($512 \to 1$)
Total parameters	5.9 million

Table S25: Hanabi belief network architecture.

**Hyperparameter**	**Value**
Observation encoder	2-layer MLP (hidden dim 512, ReLU)
Sequence	2-layer LSTM (hidden dim 512)
Autoregressive card prediction – Embedding	Linear ($25 \to 64$, no bias)
Autoregressive card prediction – LSTM	1-layer LSTM (input dim 576, hidden dim 512)
Autoregressive card prediction – Output	Linear ($512 \to 25$)
Total parameters	7.05–7.37 million

S6.4 Search

For the search, we ran $10{,}000$ simulations at each search step, evenly distributed across all legal actions. Let A denote the set of legal actions. We sampled $\frac{10{,}000}{|A|}$ hands from the belief model and used the same sampled hands for all legal actions during search. We rolled out until the end of the episode to collect cumulative return.

Table S26: Hanabi search hyperparameters.

**Hyperparameter**	**Value**
Number of rollouts	$10{,}000$
Update step size	30
Rollout temperature	0

S7 *Dou Dizhu* Implementation Details

The policy network was trained for 2 days on 4 H100s. The belief network was trained for 8 hours on 4 H100s.

S7.1 Actor Learning

Table S27: *Dou Dizhu* actor learning hyperparameters.

**Hyperparameter**	**Value**
Importance ratio clipping parameter	$0.02 \cdot \min\left\{\frac{5000}{\text{Training iteration number}}, 1\right\}$
Exponential moving average smoothing factor	0.999
Advantage estimation λ	0.95
Outcome estimation λ	0.95
Reverse KL to data collection policy loss coefficient	0
Muon learning rate	$4 \times 10^{-4} \cdot \min\left\{\frac{5000}{\text{Training iteration number}}, 1\right\}$
Regularization temperature	$0.1 \cdot \min\left\{\frac{500}{\text{Training iteration number}}, 1\right\}^{0.5}$
Maximum gradient norm	1
Number of epochs per training iteration	4
Value loss coefficient	0.5
Number of parallel environments	512
Trajectories collected per iteration	2,048
Batch size	512

S7.2 Belief Learning

In *dou dizhu*, the hidden state consists of the unseen cards held by the other two players. We therefore trained a belief model that autoregressively predicts card ownership in descending rank order. At each prediction step, the model takes the available observation history and the previously predicted belief tokens as input, and predicts which player holds the next card. The belief model is an autoregressive transformer: each historical game state is encoded by a small MLP and projected to the transformer embedding dimension, while each belief token is represented as the sum of a card embedding and a player embedding. We trained the model from snapshots collected at each

time step, using an NLL loss on the belief-token targets. During online search, the model is queried autoregressively to sample or score hidden-card assignments. We visualize the training dynamics in Supplementary Figure S7; the hyperparameters are listed below.

Table S28: *Dou dizhu* belief learning hyperparameters.

**Hyperparameter**	**Value**
Muon [60] learning rate	4×10^{-4}
WSD scheduler stable ratio	0.9
Maximum gradient norm	1.0
Time steps collected per iteration	2,048

S7.3 Networks

Table S29: *Dou dizhu* actor network information.

**Hyperparameter**	**Value**
Transformer depth	4
Embedding dimension	128
Number of heads	4
Feedforward depth	3
Feedforward dimension	256
Total parameters	1.9 million

Table S30: *Dou Dizhu* belief network information.

**Hyperparameter**	**Value**
Transformer depth	4
Embedding dimension	256
Number of heads	4
Feedforward depth	2
Feedforward dimension	256
Total parameters	3.3 million

S7.4 Search

Due to the large action space in *dou dizhu*, Ataraxos decomposes each action into a sequence of smaller decisions [61]. Rather than selecting a full action in one step, the policy incrementally

generates an action-description prefix: at each step, it chooses one valid option to append to the current prefix. Ataraxos applies search at each of these intermediate decision points, evaluating the valid continuations conditioned on the current prefix. Let A denote the set of valid options at the current step. For each option, the search procedure samples $\lceil 200/|A| \rceil$ hidden hands from the belief model. It then estimates the value of that option by forcing the option to be taken under each sampled hand and rolling out the policy network until the end of the game to collect the terminal rewards. The final choice is obtained by taking one damped policy-improvement step from the policy network, with update step size 5.

Table S31: *Dou dizhu* search hyperparameters.

**Hyperparameter**	**Value**
Number of rollouts	200
Update step size	5
Rollout temperature	0

S8 Hanabi Score Details

This section provides the detailed Hanabi evaluation numbers underlying the results reported in the Methods.

Table S32: Performance of Ataraxos on Hanabi variants with different numbers of players. We evaluate Ataraxos with different numbers of search players in each variant. Entries report means $\pm$ standard errors over $10{,}000$ games per condition.

No. of players	No. of search players	Score	Perfect
2	0	24.454 ± 0.008	$69.58\% \pm 0.33\%$
	1	24.614 ± 0.008	$75.72\% \pm 0.43\%$
	2	24.654 ± 0.007	$77.53\% \pm 0.42\%$
3	0	24.612 ± 0.008	$77.61\% \pm 0.29\%$
	1	24.780 ± 0.006	$84.77\% \pm 0.36\%$
	2	24.837 ± 0.005	$87.81\% \pm 0.33\%$
	3	24.863 ± 0.005	$89.90\% \pm 0.30\%$
4	0	24.466 ± 0.008	$68.52\% \pm 0.33\%$
	1	24.683 ± 0.007	$77.65\% \pm 0.42\%$
	2	24.776 ± 0.006	$83.50\% \pm 0.37\%$
	3	24.821 ± 0.005	$86.13\% \pm 0.35\%$
	4	24.852 ± 0.005	$88.40\% \pm 0.32\%$
5	0	23.485 ± 0.012	$26.81\% \pm 0.31\%$
	1	24.008 ± 0.011	$39.19\% \pm 0.49\%$
	2	24.213 ± 0.010	$47.43\% \pm 0.50\%$
	3	24.340 ± 0.009	$53.94\% \pm 0.50\%$
	4	24.393 ± 0.009	$56.84\% \pm 0.50\%$
	5	24.410 ± 0.009	$58.05\% \pm 0.49\%$

S9 *Dou Dizhu* Additional Results

This section provides the full role-averaged performance matrix (Table S33), the performance breakdown by game role (Table S34), training diagnostics for the *dou dizhu* belief model (Figure S7), and the sweep over update sizes (Figure S8).

Table S33: *Dou dizhu* role-averaged performance. Entries report means ± standard errors. Comparisons involving Ataraxos use 10,000 duplicated deals; other comparisons use 100,000 duplicated deals.

Row vs Col	Ataraxos	Policy Network	PerfectDou	DouZero
Ataraxos	—	0.107 ± 0.012	0.199 ± 0.015	0.350 ± 0.016
Policy Network	-0.107 ± 0.012	—	0.152 ± 0.004	0.286 ± 0.004
PerfectDou	-0.199 ± 0.015	-0.152 ± 0.004	—	0.142 ± 0.004
DouZero	-0.350 ± 0.016	-0.286 ± 0.004	-0.142 ± 0.004	—

The entries in Table S33 not involving Ataraxos (i.e., those among the policy network, PerfectDou, and DouZero) are taken from prior evaluations using 100,000 duplicated deals.

We further report performance broken down by role in Table S34, where the row agent controls the two independent peasants and the column agent plays as the landlord. Comparing Ataraxos with the policy network shows that the search routine provides particularly large gains on the peasant side. This suggests that the search is especially useful for improving coordination between the two cooperating peasant agents.

Table S34: Performance broken down by game role. The row agent is the peasants; the column agent is the landlord. Entries report means ± standard errors. Comparisons involving Ataraxos use 10,000 duplicated deals; other comparisons use 100,000 duplicated deals.

P \ L	Ataraxos	Policy Network	PerfectDou	DouZero
Ataraxos	0.696 ± 0.035	0.827 ± 0.035	0.855 ± 0.034	1.003 ± 0.034
Policy Network	0.612 ± 0.035	0.707 ± 0.011	0.811 ± 0.011	0.877 ± 0.011
PerfectDou	0.456 ± 0.036	0.507 ± 0.011	0.601 ± 0.011	0.656 ± 0.011
DouZero	0.301 ± 0.037	0.304 ± 0.011	0.373 ± 0.011	0.453 ± 0.011

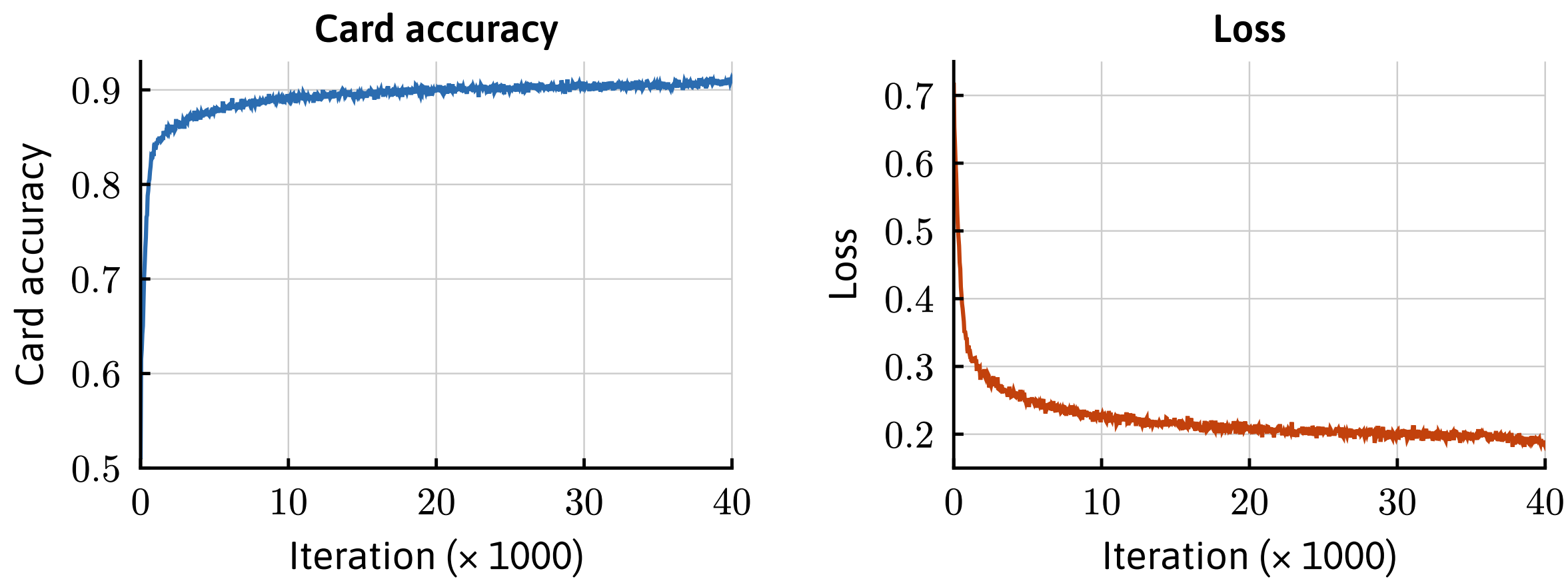

Figure S7: *Dou dizhu* belief-model training dynamics. Card-ownership accuracy (left) is the fraction of autoregressive hidden-card predictions for which the model identifies the ground-truth holder of the card. Loss (right) is the corresponding mean negative log-likelihood assigned to the ground-truth holder of the card. Both metrics are averaged over valid hidden-card predictions within each belief sequence and then over sequences.

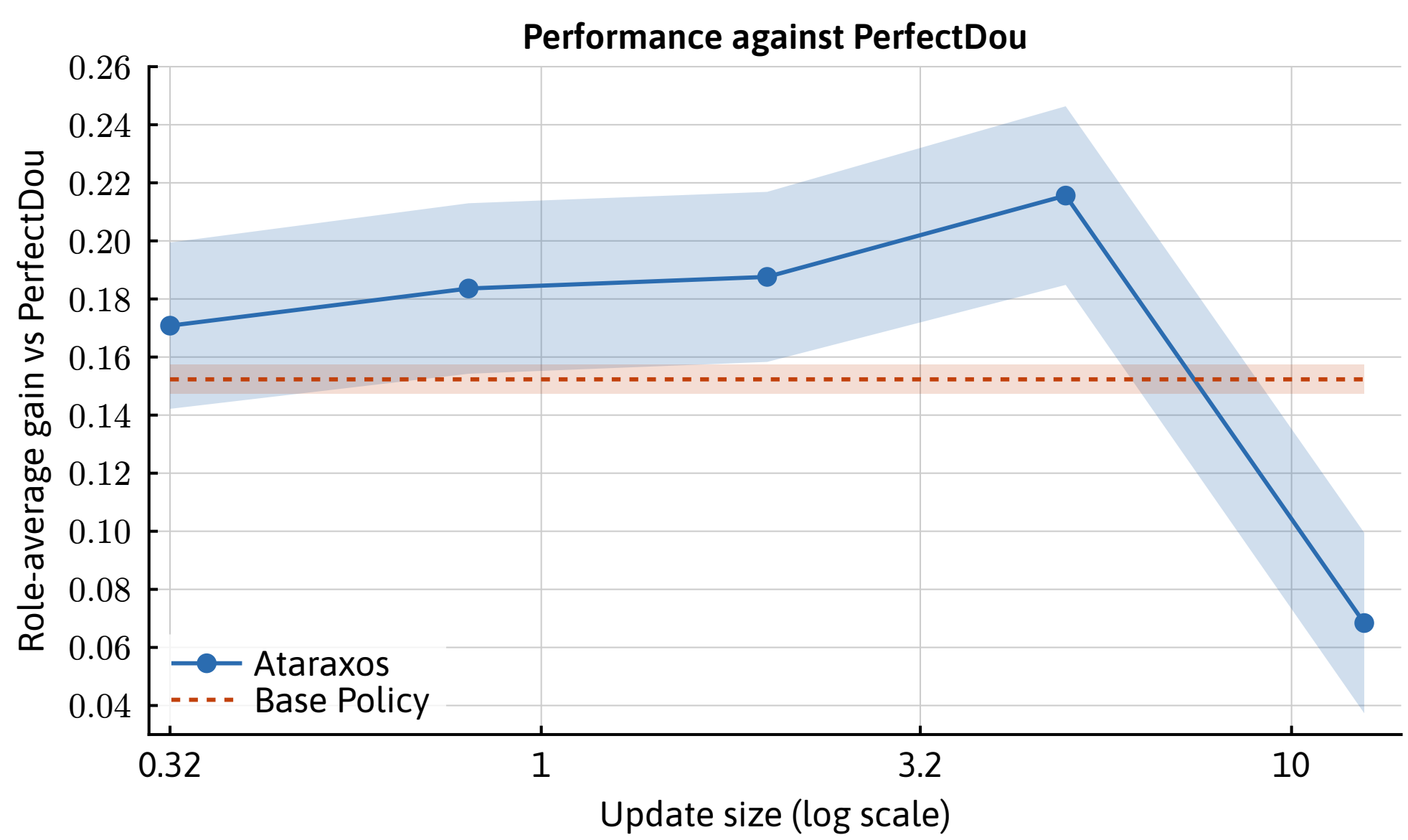

Figure S8: Role-averaged score of Ataraxos against PerfectDou under varying search update step sizes. Each setting was evaluated over $2{,}500$ duplicated deals. The dashed line shows the performance of the policy network (i.e., Ataraxos without search).

Supplementary References

[56] Jimmy Lei Ba, Jamie Ryan Kiros, and Geoffrey E. Hinton. Layer normalization, 2016. URL `https://arxiv.org/abs/1607.06450`.

[57] Kaiming He, Xiangyu Zhang, Shaoqing Ren, and Jian Sun. Deep residual learning for image recognition. In *Conference on Computer Vision and Pattern Recognition (CVPR)*, 2016.

[58] Kaiming He, Xiangyu Zhang, Shaoqing Ren, and Jian Sun. Delving deep into rectifiers: Surpassing human-level performance on ImageNet classification. In *International Conference on Computer Vision (ICCV)*, 2015.

[59] Hengyuan Hu, Adam Lerer, Noam Brown, and Jakob N. Foerster. Learned belief search: Efficiently improving policies in partially observable settings, 2021. URL `https://arxiv.org/abs/2106.09086`.

[60] Keller Jordan, Yuchen Jin, Vlado Boza, Jiacheng You, Franz Cesista, Laker Newhouse, and Jeremy Bernstein. Muon: An optimizer for hidden layers in neural networks, 2024. URL `https://kellerjordan.github.io/posts/muon/`.

[61] Zhiyuan Fan and Gabriele Farina. GAE falls short in imperfect-information self-play reinforcement learning, 2026. URL `https://arxiv.org/abs/2605.19235`.
