# Supplementary material for: Scalable decision-making for games of imperfect information
Source: Nature. 2026 Sep 30;658(8134):55–9. doi: 10.1038/s41586-026-11036-y (PMC13626944; doi:10.1038/s41586-026-11036-y)
Supplement: Supplementary file 2 [file 41586_2026_11036_MOESM2_ESM.pdf]

nature portfolio

Peer Review File

Scalable Decision Making for Games of Imperfect Information

Corresponding Author: Professor Gabriele Farina

Any redactions in this file are there to maintain patient confidentiality, the confidentiality of unpublished data, or to remove third-party material.

This file contains all reviewer reports in order by version, followed by all author rebuttals in order by version.

Version 0:

Reviewer comments:

Referee #1

(Remarks to the Author)
Summary of key result:
This paper introduces Ataraxos, the AI system to achieve superhuman performance in Stratego, a game with massive hidden information and an enormous configuration space. This resolves a long-standing open challenge in imperfect-information game AI and represents a major empirical milestone. Notably, this level of performance was achieved at a relatively low computational cost, further strengthening the significance of the result.

Originality and significance:
The introduction of a dedicated belief network to predict hidden opponent pieces is a key architectural contribution. This explicit modeling of uncertainty enables effective decision-making and makes test-time search feasible without belief-tree enumeration.
Unlike AlphaZero-style methods, the system does not use search during self-play training, avoiding instability and high computational cost in hidden-information settings. Despite this departure from conventional practice, the method still achieves superhuman performance.
Search is reintroduced at test time via a simple update-equivalence formulation, leading to consistent performance gains with limited additional computation. This provides an elegant bridge between learning and planning.
Another technical contribution is a dynamically damped self-play framework that coordinates regularization strength, policy update size, and policy strength to stabilize learning under hidden information.
Evaluation against a real, adaptive human expert, this is far stronger than typical game AI evaluations.

Data & methodology:
The validation of the trained model's performance against human champions is reasonably strong and convincing. However, the presentation of the model architecture and methodology lacks clarity. In particular, the description of how each component is trained, and how it is subsequently used is not sufficiently clear. This issue is especially pronounced for the belief network, whose role and training procedure are not explained in enough detail.

Appropriate Use of Statistics and Treatment of Uncertainty
The paper provides a generally appropriate and transparent statistical treatment of its evaluation. The authors report the full 20-game match results, define effective win rate clearly, and provide a p-value under an explicit i.i.d. assumption. They also acknowledge that this independence assumption is likely violated due to human adaptation, which is a responsible treatment of uncertainty. Confidence intervals are shown for Elo under different search settings

Suggested improvements:

The writing of the paper needs improvement. In particular, the sentence formulation and overall structure lack sufficient clarity and signposting, which makes it unnecessarily difficult for readers to follow and understand the technical details.
The belief network is an important component of the model, yet its introduction and training procedure are not presented with sufficient clarity. The description of how it is trained is given only in very brief sentences and is interwoven within the test-time section, alongside explanations of how it is used during testing. This mixing of training-time and test-time details makes it difficult to clearly understand how the belief network functions in each phase and how the two roles differ.

Moreover, important details about the training process of the belief network appear to be missing in the paper. The training procedure is only briefly referenced in Appendix 5 ("Belief Learning," lines 638–640), which is insufficient for readers to fully understand or reproduce this component of the method.
To better highlight the true novelty and primary contribution of the paper, it would be helpful to clarify the main source of its reported low training cost. Specifically, it is unclear whether the model achieves strong performance with significantly fewer samples (e.g., fewer self-play games or simulations), indicating improved sample efficiency, or whether the reduced cost is mainly due to engineering optimizations that make the training process more computationally efficient. At present, the paper does not clearly distinguish between these two possibilities. While both would be valuable contributions, they imply very different types of advances. Improved sample efficiency would represent a stronger algorithmic contribution, whereas cost reduction primarily driven by systems-level optimization would reflect an engineering achievement. In my view, the former would be more conceptually impressive. Clarifying which factor plays the dominant role would help readers better understand the paper's true contribution.
A central design decision of the paper is to avoid search during self-play training, despite extensive prior success of search-based self-play methods in game AI. The authors state that: "We experimented with search-based self-play methods, but found that the slowdown on data generation imposed by the search methods we tried outweighed potential benefits." However, the paper does not present any explicit experimental results, ablation studies, or quantitative comparisons supporting this claim. Specifically:
No performance curves comparing training with and without search are provided.
No measurements of data-generation throughput, sample efficiency, or wall-clock performance are reported.
No details are given regarding the search variants evaluated (e.g., depth, rollout count, belief handling).
As a result, the claim that search-based self-play is ineffective during training remains asserted rather than demonstrated.
The system combines many techniques at once:
dynamic KL regularization
learning-rate schedules
advantage filtering
entropy annealing
belief networks
EMA parameters
update-equivalence search
There is no component-wise ablation answering:
Which mechanisms are essential?
Which are merely helpful?
Which compensate for others?
This makes the system hard to reason about scientifically and hard to reproduce or simplify.

line 73-75:The paper does not provide an explicit or formal definition of "strong policy" versus "weak policy," which may lead to ambiguity. For instance, the statement "adapting both quantities to policy strength creates rapid improvements when the policy is weak and continued improvement when the policy is strong—avoiding both update sizes so small as to make improvements impractical and regularization so large as to compromise the policy" relies on an undefined notion of policy strength. The same issue arises throughout the paper wherever policy strength is referenced without formal clarification.

line 100: "but found that the slowdown on data generation imposed by the search methods we tried outweighed potential benefits."
-> "but the slowdown in data generation imposed by these search methods outweighed the potential benefits."

Lines 112–115: The logic here is unclear. The text states that The superiority of Monte Carlo returns for advantage estimation is unusual in reinforcement learning, but has also been observed in language model reasoning, possibly suggesting that this superiority is related to the bandit-like structure shared by the two problems. If this superiority were indeed driven by a shared bandit-like structure, one would expect it to be more common in reinforcement learning settings exhibiting such structure. As written, the conclusion does not seem to follow from the preceding claims.

Line 121: The statement "Ataraxos incorporates additional terms into the losses of its networks" is unclear, as the losses being referenced are never explicitly defined in the paper. It is therefore ambiguous which objective(s) the regularization terms are added to.

Conclusion:
This work presents an impressive empirical result achieved at relatively low cost, and the validation against human champions is convincing. However, beyond the headline result, the paper would benefit from clearer exposition and additional analysis to better articulate its true novelty and the precise nature of the progress it represents, as discussed above.

References: appropriate credit to previous work?
Yes

Clarity and context:
Writing in abstract, introduction and conclusions are all good.

(Remarks on code availability)

Referee #2

(Remarks to the Author)
A. Summary of the key results

This work focuses on Stratego, a board game with hidden information, and proposes Ataraxos, an optimized method based on self-play reinforcement learning and test-time search. Compared with existing approaches, Ataraxos achieves significant improvements in training time and computational cost, reaching a high level of performance within only a few days of training using 16 H100s. In matches against a human expert Pim, Ataraxis demonstrates strong ability, achieving 15 wins, 1 loss, and 4 draws over 20 games.

B. Originality and significance

As discussed by the authors, prior work has already explored strategies for games with hidden information, with the most representative study on Stratego being DeepNash, completed by the DeepMind team in 2022. DeepNash similarly applies self-play–based reinforcement learning to Stratego and achieves strong performance against both other AI agents and human players. Therefore, from the perspective of the research problem, this work does not introduce or investigate a fundamentally new problem.

In terms of methodology, the proposed approach adopts a framework based on reinforcement learning and search, which has been widely used in board-game domains (including earlier systems such as AlphaGo in perfect information settings). Compared with DeepNash, a major difference lies in the fact that the authors do not adopt an end-to-end modeling paradigm. Instead, they train two separate models for the setup phase and the action phase. The authors argue that this design “avoids trade-offs involving design choices, network architecture, and hyperparameter regimes”. However, such a separation requires additional effort in coordinating and optimizing the jointly trained models compared with an end-to-end approach.

Regarding training efficiency, Ataraxos achieves substantial improvements over DeepNash and related work, benefiting from a series of carefully designed optimizations in the training process. This improvement in efficiency constitutes the key technical innovation and primary contribution of the manuscript.

C. Data & methodology

The proposed method does not rely on existing human game play data but instead adopts a self-play strategy to generate training data. As reported in Table 27, approximately 163 million self-play games are generated during training.

In the method part, the authors make effective use of established technical approaches and provide a clear description of the key procedures for both the setup and action phases. Several efficiency-oriented optimization strategies are incorporated, which help readers clearly understand the underlying design choices and training process. However, from a methodological perspective, this study appears to primarily integrate and refine existing techniques, rather than introducing substantially new technical innovations in the method itself.

D. Appropriate use of statistics and treatment of uncertainties

I consider the experimental part to be one of the weaknesses of this work. The current experiments mainly report two sets of quantitative results: one consists of 20 games against the top human player Pim (15 wins, 1 loss, and 4 draws), and the other reports results against a subset of challengers at the 2025 Stratego World Championship, with 38 wins and 2 losses (the exact number of participants is unclear).

The scale of these evaluation experiments is relatively limited, making it difficult to provide sufficiently robust quantitative evidence to convincingly demonstrate the model’s advantages and distinctive characteristics. Although the authors mention in the Appendix that DeepMind currently cannot support a direct comparison between DeepNash and Ataraxos, it would still be valuable to include additional evaluations. For example, DeepNash compared its performance against a variety of other Stratego agents through extensive matches, and similar large-scale evaluations here could help better substantiate the effectiveness of the proposed strategy.

E. Conclusions

The conclusions are reasonable. The margin of victory (15 wins vs 1 loss) leaves little doubt regarding the "superhuman" claim. The authors properly contextualize their work against DeepNash in the appendix, explaining why a direct head-to-head comparison was impossible (DeepNash code availability) and comparing costs instead.

F. Suggested improvements

I believe the following issues (some of which are suggestions) still need to be addressed in this work:

1. In the description of the Ataraxos method, the authors should provide a more explicit comparison with end-to-end approaches, discussing the respective advantages and disadvantages. In addition, the efficiency discussion section could include a concise explanation of the core factors contributing to the observed efficiency gains (e.g., reduced parameter scale, optimized training pipelines, or improved inference strategies).

2. This manuscript would benefit from more experimental evaluations, even against earlier or weaker Stratego agents. As the authors themselves note, in hidden-information games such as Stratego, even high-level players may occasionally defeat top experts. Therefore, larger-scale quantitative evaluations are necessary to robustly demonstrate performance advantages.

3. The current training appears to be based mainly on loss convergence and wall-clock training time. While Elo-based evaluations indicate continuous performance improvements, it would be valuable to further analyze training stability by presenting more comparisons for analysis, such as the win rate of a model trained for 2000 H100-hours versus one trained for 1000 H100-hours.

4. The setup phase of a match represents a crucial decision-making process, and the authors train a setup network for this stage. Although this manuscript reports the optimal probability distribution over piece placements and presents the setups used in the 20 games against Pim, it remains unclear how the final setup is selected in practice. Specifically, does this process rely on additional prior information, or is the setup autonomously chosen by the system without user intervention?

5. The scalability and generalizability of the proposed technique are discussed only briefly. In particular, its applicability to other tasks beyond Stratego remains unclear. This issue is closely tied to the methodological novelty of the work: if the proposed approach is heavily optimized for a specific task, its broader impact and originality may be limited.

G. References

The references are appropriate. The paper correctly cites foundational work in transformers, RL for games (AlphaZero, DeepStack), and the specific predecessor DeepNash.

H. Clarity and context

The manuscript is lucid. The abstract clearly states the achievement. The introduction effectively frames the difficulty of hidden information. The distinction between the training phase (filtering, damping) and the test-time phase (search via update equivalence) is structurally clear. The appendices provide sufficient detail for replication, particularly regarding the simulator design.

(Remarks on code availability)
The code provides enough instructions for installing and running the application. I was able to install and run the code. I did not have time to re-run the entire experiments and reproduce the results of the paper.

Referee #3

(Remarks to the Author)
I co-reviewed this manuscript with one of the reviewers who provided the listed reports.

(Remarks on code availability)

Version 1:

Reviewer comments:

Referee #1

(Remarks to the Author)
The comments have been all addressed.

(Remarks on code availability)
The code is too involved. Cannot review code in the timeframe.

Referee #2

(Remarks to the Author)
This work focuses on Stratego, a board game with hidden information, and proposes Ataraxos, an optimized method based on self-play reinforcement learning and test-time search. Compared with existing approaches, Ataraxos achieves significant improvements in training time and computational cost, reaching a high level of performance within only a few days of training using 16 H100s. In matches against a human expert Pim, Ataraxis demonstrates strong ability, achieving 15 wins, 1 loss, and 4 draws over 20 games. The experiments on the other three games demonstrate that the proposed method has strong generalization capability.

1. Originality and Significance
As discussed by the authors, prior work has already explored strategies for games with hidden information, with the most representative study on Stratego being DeepNash, completed by the DeepMind team in 2022. DeepNash similarly applies self-play–based reinforcement learning to Stratego and achieves strong performance against both other AI agents and human players. Therefore, from the perspective of the research problem, this work does not introduce or investigate a fundamentally new problem.

In terms of methodology, the proposed approach adopts a framework based on reinforcement learning and search, which has been widely used in board-game domains (including earlier systems such as AlphaGo in perfect information settings). Compared with DeepNash, a major difference lies in the fact that the authors do not adopt an end-to-end modeling paradigm. Instead, they train two separate models for the setup phase and the action phase. The authors argue that this design “avoids trade-offs involving design choices, network architecture, and hyperparameter regimes”.

In terms of training efficiency, Ataraxos achieves substantial improvements over DeepNash and related work, benefiting from a series of carefully designed optimizations in the training process. This improvement in efficiency constitutes the key technical innovation and primary contribution of the manuscript.

2. Data and Methofology

The proposed method does not rely on existing human game play data but instead adopts a self-play strategy to generate training data. As reported in Table 27, approximately 163 million self-play games are generated during training.

In the method part, the authors make effective use of established technical approaches and provide a clear description of the key procedures for both the setup and action phases. In particular, several efficiency-oriented optimization strategies are incorporated, which help readers clearly understand the underlying design choices and training process. However, from a methodological perspective, this study appears to primarily integrate and refine existing techniques, rather than introducing substantially new technical innovations in the method itself.

3. Experiments

The experiments mainly report two sets of quantitative results: one consists of 20 games against the top human player Pim (15 wins, 1 loss, and 4 draws), and the other reports results against a subset of challengers at the 2025 Stratego World Championship, with 38 wins and 2 losses (the exact number of participants is unclear).
In the revised version, the authors conducted two additional sets of experiments. First, the results against earlier AI bots from the Australian team show that the proposed model achieved a win rate close to 100%. In addition, the authors compared DeepNash’s performance at the 2023 Stratego World Championship with the performance of the proposed model in 2025, which further demonstrates the improvement in model capability.

4. Suggestions

The authors have addressed several limitations and concerns I raised regarding this work in the revision and have provided targeted responses and additional experiments. In particular, they clarified important details of the model, added analytical experiments, and further demonstrated the generality of the method by applying it to additional tasks and achieving strong results.
These responses and newly added contents adequately address the identified weaknesses and provide stronger support for the novelty and potential impact of the paper. Therefore, I have no further comments or suggestions.

5. Conclusion

Overall, this manuscript is clearly presented and demonstrates good performance. The authors conducted analytical experiments and generalization experiments, demonstrating that the method has promising application prospects. I believe this study has meaningful impact and potential.

(Remarks on code availability)

Referee #3

(Remarks to the Author)

I co-reviewed this manuscript with one of the reviewers who provided the listed reports.

(Remarks on code availability)

Open Access This Peer Review File is licensed under a Creative Commons Attribution 4.0 International License, which permits use, sharing, adaptation, distribution and reproduction in any medium or format, as long as you give appropriate credit to the original author(s) and the source, provide a link to the Creative Commons license, and indicate if changes were made.
In cases where reviewers are anonymous, credit should be given to 'Anonymous Referee' and the source.
The images or other third party material in this Peer Review File are included in the article's Creative Commons license, unless indicated otherwise in a credit line to the material. If material is not included in the article's Creative Commons license and your intended use is not permitted by statutory regulation or exceeds the permitted use, you will need to obtain permission directly from the copyright holder.
To view a copy of this license, visit https://creativecommons.org/licenses/by/4.0/

May 26, 2026

RE: Response to Reviewers, manuscript `2025-11-30735`

We thank the editor and reviewers for their constructive feedback. We have substantially revised the manuscript in response, including evaluating our methodology for three new games (with new human experiment data), spanning competitive, cooperative, and mixed competitive-cooperative games. In all domains, we achieved state-of-the-art results at moderate costs.

---

1. Response to the Editor

> **Re**: Better characterize conceptual advancement and broad applicability

We have taken two concrete steps to address this concern in the revision.

First, we made the algorithmic nature of the advance explicit. The revised *Training Information* section (§1.7) now directly compares our training run against DeepNash along three axes: compute cost, number of self-play games, and number of training examples. *Our training run consumed less than 1/30th of the self-play games and less than 1/100th of the training examples.* The dramatic improvement in performance compared to DeepNash, while using two orders of magnitude fewer training examples indicates a large algorithmic/conceptual advance rather than engineering optimization. (Implementation speed has no bearing on the number of samples necessary for learning.)

Second, we added results showing that the same techniques achieve similarly state-of-the-art performance on three additional imperfect-information games—Barrage Stratego, Hanabi, and *dou dizhu*—spanning adversarial, cooperative, and two-team zero-sum settings.

---

2. Response to Referee 1

2.1. Presentation of methodology and components

> **Re**: However, the presentation of the model architecture and methodology lacks clarity. In particular, the description of how each component is trained, and how it is subsequently used is not sufficiently clear. This issue is especially pronounced for the belief network, whose role and training procedure are not explained in enough detail.

> **Re**: [..] important details about the training process of the belief network appear to be missing in the paper. The training procedure is only briefly referenced in Appendix 5 ("Belief Learning," lines 638–640), which is insufficient for readers to fully understand or reproduce this component of the method.

We revised the organization of the main text and clarified several passages in the narrative. In particular, we separated belief modeling from test-time search into distinct subsections (now §1.5 and §1.6), added explicit text stating that self-play games are generated by directly sampling from the networks (§1.3), added explicit text stating that the setups deployed at test time are directly sampled from the setup network (§1.6), and improved the wording around dynamic damping and search (§1.4-1.6).

We added a dedicated *Belief Modeling* subsection (§1.5) to the main text, and expanded the corresponding *Belief Learning* appendix section (§D.5). The revised text in §1.5 and §1.6 now separates the two roles of the belief network:

- *Training* of the belief network: the belief network is trained with teacher forcing on every position of trajectories sampled from the final self-play policy, with the objective of maximizing the log likelihood of the true types of the opponent's hidden pieces given the information available to the player at that position. The appendix specifies that training uses every position from these trajectories with no filtering or subsampling (§D.5).
- *Use* of the belief network: at test time, the belief network is queried prior to each move to sample a collection of possible game states given the current position; these states seed the subsequent rollout-based search.

2.2. Source of low cost

> **Re**: To better highlight the true novelty and primary contribution of the paper, it would be helpful to clarify the main source of its reported low training cost. Specifically, it is unclear whether the model achieves strong performance with significantly fewer samples (e.g., fewer self-play games or simulations), indicating improved sample efficiency, or whether the reduced cost is mainly due to engineering optimizations that make the training process more computationally efficient. [..] Clarifying which factor plays the dominant role would help readers better understand the paper's true contribution.

We made the algorithmic nature of the advance explicit. Specifically, the revised *Training Information* section (§1.7) now directly compares our training run against DeepNash along three axes: compute cost, number of self-play games, and number of training examples. Our training run consumed roughly 1/500th of the compute cost, less than 1/30th of the self-play games, and less than 1/100th of the training examples. The dramatic improvement in performance compared to DeepNash, while using 2 orders of magnitude fewer training examples indicates a large algorithmic/conceptual advance rather than engineering optimization alone. Please see also the response to the Editor.

2.3. Self-play search

> **Re**: A central design decision of the paper is to avoid search during self-play training, despite extensive prior success of search-based self-play methods in game AI. The authors state that: "We experimented with search-based self-play methods, but found that the slowdown on data generation imposed by the search methods we tried outweighed potential benefits." However, the paper does not present any explicit experimental results, ablation studies, or quantitative comparisons supporting this claim. [..]

To clarify, the claim in the paper was not that search-based self-play cannot be made to work, only that, as quoted above, we did not observe a benefit from the methods we tried. However, this may have been due to our limited compute budget target (most of our development experiments were performed on a single GPU). It may also be the case that other methods that we did not try would have been superior. Since this point could be read more broadly and was not central to the paper, we removed the sentence from the revised manuscript.

2.4. Ablations

> **Re**: The system combines many techniques at once: [..] There is no component-wise ablation answering: Which mechanisms are essential? Which are merely helpful? Which compensate for others? This makes the system hard to reason about scientifically and hard to reproduce or simplify.

We added several analyses to make the contributions of the main components more explicit:

- An ablation figure (Figure 15) in the *Other Experiments* section (Appendix §F) of the supplement evaluates the effect of removing learning-rate annealing, advantage filtering, and regularization annealing (entropy/KL-to-magnet) in turn; all three materially affected performance over the course of training. The regularization annealing (entropy/KL-to-magnet) ablation specifically tests the effect of annealing the regularization coefficient—the maximum entropy term for setups and the reverse KL-to-magnet penalty for moves.
- The search hyperparameter table (Table 29) includes a belief-network ablation and a direct comparison between the search policy and the raw policy network, isolating the contribution of search over the baseline policy.
- Finally, Figure 16 compares current iterates against their exponential moving averages across three seeds against a fixed reference model, showing that exponential moving averaging reduces performance variance.

Also note Figure 14 from the original submission, which ablates distributed training, setup learning, advantage filtering, and use of the `bfloat16` data type.

We have also analyzed training data to assess training stability (see also the *Training Stability* subsection in Referee 2's answer). In Figure 13, we provide a pairwise checkpoint comparison analysis. We evaluated 22 checkpoints from the training run—selecting those whose iteration indices were divisible by 2,000—against each other over 2,000 games per pair. The resulting score matrix shows that later checkpoints generally outperform earlier ones, consistent with approximately monotonic improvement over the course of training.

2.5. Miscellaneous feedback on wording and clarity

Thank you for the feedback.

> **Re**: line 73-75: The paper does not provide an explicit or formal definition of "strong policy" versus "weak policy," which may lead to ambiguity.

We removed that terminology from the key explanatory passage and throughout the manuscript, and now describe the schedule in terms of early versus late training.

> **Re**: line 100: [..]

Thank you. As discussed in "2.3. Self-play search" above, we have reworked the section and removed the sentence to which this comment was referring.

> **Re**: Lines 112–115: The logic here is unclear. The text states that The superiority of Monte Carlo returns for advantage estimation is unusual in reinforcement learning, but has also been observed in language model reasoning, possibly suggesting that this superiority is related to the bandit-like structure shared by the two problems.

The intended logic was based on the premise that there are relatively few large-scale bandit-like environments that are approached using deep reinforcement learning. Since the connection was not a substantive claim of the paper and risked confusion, we removed the inference about shared bandit-like structure and retained only the empirical observation.

> **Re**: Line 121: The statement "Ataraxos incorporates additional terms into the losses of its networks" is unclear, as the losses being referenced are never explicitly defined in the paper.

We clarified this in the revised manuscript. The passage now explicitly directs the reader to the *Setup Learning* and *Move Learning* appendix sections §D.3 and §D.4, where the objectives are defined in full and the regularization terms are identified in each case.

---

3. Response to Referee 2

3.1. Novelty of research problem

> **Re**: from the perspective of the research problem, this work does not introduce or investigate a fundamentally new problem.

We agree that Stratego is an established benchmark; the contribution of the manuscript is not the introduction of a new problem, but a scalable approach to self-play reinforcement learning and test-time search under large amounts of hidden information.

3.2. Methodological novelty

> **Re**: from a methodological perspective, this study appears to primarily integrate and refine existing techniques, rather than introducing substantially new technical innovations in the method itself.

Our methodology for search under massive imperfect information is new, and addresses a long-standing desideratum in the field. DeepNash (DeepMind, 2022) expressly left open the question of search at this scale. As discussed in the paper, prior techniques (such as those based on public belief states and used in landmark AI for poker) were inapplicable since they require enumeration of the possible underlying hidden states, which is impossible in settings like Stratego.

In addition, our dynamic damping methodology is also new in the context of deep RL.

We also remark that these techniques demonstrate success in what is currently the largest game to which imperfect-information search has been successfully applied.

In the new version, we demonstrate the broad applicability of these techniques in other challenging large-scale games with massive imperfect information, including adversarial, cooperative, and mixed cooperative-competitive games (§3). We achieved state-of-the-art performance in all of them, at very modest costs.

3.3. Evaluation section

> **Re**: I consider the experimental part to be one of the weaknesses of this work. The current experiments mainly report two sets of quantitative results: one consists of 20 games against the top human player Pim (15 wins, 1 loss, and 4 draws), and the other reports results against a subset of challengers at the 2025 Stratego World Championship, with 38 wins and 2 losses (the exact number of participants is unclear) [..] The scale of these evaluation experiments is relatively limited, making it difficult to provide sufficiently robust quantitative evidence to convincingly demonstrate the model's advantages and distinctive characteristics. Although the authors mention in the Appendix that DeepMind currently cannot support a direct comparison between DeepNash and Ataraxos, it would still be valuable to include additional evaluations. For example, DeepNash compared its performance against a variety of other Stratego agents through extensive matches, and similar large-scale evaluations here could help better substantiate the effectiveness of the proposed strategy. [..] This manuscript would benefit from more experimental evaluations, even against earlier or weaker Stratego agents. As the authors themselves note, in hidden-information games such as Stratego, even high-level players may occasionally defeat top experts. Therefore, larger-scale quantitative evaluations are necessary to robustly demonstrate performance advantages.

We agree that evaluation scale is important, and we have added several additional evaluations in the revision. At the same time, the 20-game series against Pim Niemeijer was already unusually large for an elite human Stratego evaluation: it required three weeks to complete, was long enough for Pim to study and exploit a fixed Ataraxos policy, and still produced a 15-1-4 result. Under the simplifying i.i.d. calculation reported in the manuscript, this result is statistically significant; furthermore, the margin was regarded by top Stratego players as unprecedented at the highest level of play.

For context, this human evaluation was longer than several landmark human-vs-AI matches, including Deep Blue-Kasparov and AlphaGo-Lee Sedol. We nevertheless agree with the reviewer's broader point that additional quantitative evidence is valuable, and thus added the additional evaluations discussed below:

Regarding evaluations against other AI Stratego bots: the supplement now includes results against the publicly available AI bots from the University Computer Club of Australia 2012 programming competition (end of §F). The near 100% win rates indicate that these bots are not meaningful ways to measure performance.

The *Contextualization* section (§J) of the supplement also now reports that DeepNash recorded 19 wins and 9 losses at the 2023 Stratego World Championship—a significantly worse record than the 38-2 record Ataraxos achieved at the 2025 Stratego World Championship. Also, DeepNash had a negative record against the highest-ranked players who played against it [1], which included Pim Niemeijer (the expert against whom we won 15 games, lost 1, and tied 4). We report this quote from [1]:

> *"On the final day of the tournament, some of the highest ranked players that were participating in the classic tournament on the Friday and Saturday joined the battle against the AI. Most of these players won their games"*

We expanded the discussion in §J to include the above data point.

We also added evaluations for Barrage Stratego, Hanabi, and *dou dizhu* (see text under "3.8. Generalizability" in this document).

[1]: `https://www.strategonews.com/wc2023/deepnash-surprises-top-stratego-players/`

3.4. End-to-end approaches

> **Re**: In the description of the Ataraxos method, the authors should provide a more explicit comparison with end-to-end approaches, discussing the respective advantages and disadvantages.

In the revision, we rewrote the relevant passage to compare the decomposed approach directly with an end-to-end alternative (§1.1).

The revised text states that, although an end-to-end model would avoid making the phase decomposition explicit, it would require a single model and training pipeline to accommodate two structurally unrelated tasks. The subsequent sections describe the resulting design differences in detail. Among them: setup learning favored a decoder-only architecture, Monte Carlo expected return and advantage estimation, and higher learning rates and regularization temperatures, whereas move learning favored an encoder-only architecture, λ-based expected return and advantage estimators, an annealed learning-rate schedule, and lower regularization temperatures.

3.5. Source of computational efficiency

> **Re**: In addition, the efficiency discussion section could include a concise explanation of the core factors contributing to the observed efficiency gains (e.g., reduced parameter scale, optimized training pipelines, or improved inference strategies).

Concisely, the source of runtime efficiency came from a faster data collection and training pipeline. We have made this explanation more explicit, and consolidated the section within the broader discussion on engineering details in Appendix §B.

Our data collection and training pipeline is centered around our combined simulator–rollout buffer design (see Appendix §B.1), whose main merit is to save memory and reduce CPU–GPU transfers.

As we point out explicitly in §1.7, these engineering considerations are orthogonal to our technical/methodological advances, which instead drive the increase in *sample* efficiency (as opposed to *runtime* efficiency). Also, as we remark to Referee 1 and more explicitly point out in the new revision, we achieved our massive improvement in playing strength while using two-orders-of-magnitude fewer training examples.

3.6. Training stability

> **Re**: The current training appears to be based mainly on loss convergence and wall-clock training time. While Elo-based evaluations indicate continuous performance improvements, it would be valuable to further analyze training stability by presenting more comparisons for analysis, such as the win rate of a model trained for 2000 H100-hours versus one trained for 1000 H100-hours.

We added a pairwise checkpoint-comparison analysis to the supplement (Figure 13). We evaluated 22 checkpoints from the training run—selecting those whose iteration indices were divisible by 2,000—against each other over 2,000 games per pair. The resulting score matrix shows that later checkpoints generally outperform earlier ones, consistent with approximately monotonic improvement over the course of training.

3.7. Selection of the final setup

> **Re**: The setup phase of a match represents a crucial decision-making process, and the authors train a setup network for this stage. Although this manuscript reports the optimal probability distribution over piece placements and presents the setups used in the 20 games against Pim, it remains unclear how the final setup is selected in practice. Specifically, does this process rely on additional prior information, or is the setup autonomously chosen by the system without user intervention?

We clarified this in the new version. As we now mention in the *Data Generation* section (§1.3), to generate self-play games Ataraxos selects its setups and moves by directly sampling from the associated networks. The *Test-Time Search* section (§1.6) further clarifies that evaluation setups are generated the same way —by sampling directly from the setup network, with *neither search nor any other intervention applied.* The *Learned Setups* section of the supplement (§H) confirms that the setups used in the series against Pim Niemeijer were generated fully autonomously by sampling from this distribution, one per game, and displays all 20.

3.8. Generalizability

> **Re**: The scalability and generalizability of the proposed technique are discussed only briefly. In particular, its applicability to other tasks beyond Stratego remains unclear. This issue is closely tied to the methodological novelty of the work: if the proposed approach is heavily optimized for a specific task, its broader impact and originality may be limited.

We added results on three additional imperfect-information games:

- **Barrage Stratego** (adversarial): We defeated 3 separate multi-time world champions, marking the first superhuman result for the game. This is a competitively-played variant of Stratego which presents significantly different strategic challenges compared to the standard game. See §3.1.
- **Hanabi** (cooperative): We set a new state-of-the-art for 2-player, 3-player, 4-player, and 5-player Hanabi, the most competitive benchmark game for fully cooperative settings.
- ***Dou dizhu*** (team, mixed cooperative-competitive): We defeated both state-of-the-art AIs for *dou dizhu*, one of the most popular card games in China.

These tasks span adversarial, two-team zero-sum, and cooperative settings and involve different numbers of players, different action spaces, and different structures of hidden information. The fact that the same core techniques—dynamically damped self-play reinforcement learning and test-time search via update equivalence—achieve strong results across all of them supports the claim that the approach is general rather than specific to Stratego.

July 15, 2026

RE: Response to Reviewers, manuscript `2025-11-30735A-Z`

We thank the editor and reviewers for their time and constructive feedback during this process. This round of revisions had no reviewer questions to address.
